# Multivalent peptides for blood–brain barrier translocation, cell penetration and microRNA-21 inhibition in glioblastoma

**DOI:** 10.1039/d6cb00229c

**Published:** 2026-09-01

**Authors:** Talhat Chaudhry, Marco Cavaco, Vera Neves, Miguel A. R. B. Castanho, Lauren Tonge, Francesca Giuntini, Adam Schofield, Christopher R. Coxon, Kehinde Ross

**Affiliations:** a School of Pharmacy and Biomolecular Sciences, Liverpool John Moores University Liverpool UK o.k.ross@ljmu.ac.uk; b Institute for Health Research, Liverpool John Moores University Liverpool UK; c Fundação GIMM – Gulbenkian Institute for Molecular Medicine Av. Prof Egas Moniz 1649-028 Lisboa Portugal; d Faculdade de Medicina, Universidade de Lisbia Av. Prof. Egas Moniz 1649-028 Lisboa Portugal; e EaStChem School of Chemistry, The University of Edinburgh Joseph Black Building, David Brewster Road Edinburgh EH9 3FJ UK

## Abstract

Glioblastoma (GBM) is an aggressive brain cancer for which the prognosis remains poor despite the advances in our understanding of the complex heterogeneity of the disease. Therapeutic access to GBM tumours requires drugs to cross the blood–brain barrier (BBB), a physiological obstacle that restricts the passage of compounds from systemic circulation into the brain. Blood–brain barrier peptide shuttles (BBBpS), which are a subclass of cell penetrating peptides (CPPs), represent a promising approach for transferring large cargos across the BBB, especially cargos such as peptides or antisense oligonucleotides against RNA targets. Such RNA targets include microRNAs (miRNA), of which several have emerged as candidate drug targets in GBM. MicroRNA-21 is one such miRNA implicated in GBM, and although peptide-based miR-21 inhibition has been reported, delivery of peptide-based inhibitors of miR-21 across the BBB has received limited attention. Here, we selected a BBB-penetrating peptide, SYPGWSW, for conjugation to a peptide-based inihibitor of miRNA processing. We show that SYPGWSW penetrates GBM cell lines and its function depends critically on the properties of aromatic residues Tyr and Trp but not on Pro residues. Further, subsitution of Tyr with unnatural amino acids cyclohexylalanine or 1-naphthylalanine enhanced the internalisaton of the parent peptide by 2 to 4 fold. Mechanistically, the higher level of uptake was associated with enhanced stability of the peptide, which appeared to be partly associated with binding to human serum albumin. Finally we showed that a peptide inhibitor of miR-21 processing conjugated to SYPGWSW traversed an *in vitro* BBB model and enabled the functional silencing of miR-21-5p in GBM cells. Together, these findings pave the way for peptide conjugates as novel therapeutics for silencing miRNA expression in brain cancer.

## Abstract 2

We report incorporation of unnatural amino acids for improved uptake of a blood-brain barrier peptide shuttle. Fusion to a peptide-based inhibitor of miR-21 processing enabled silencing of miR-21-5p in glioblastoma cells.

## Graphical abstract

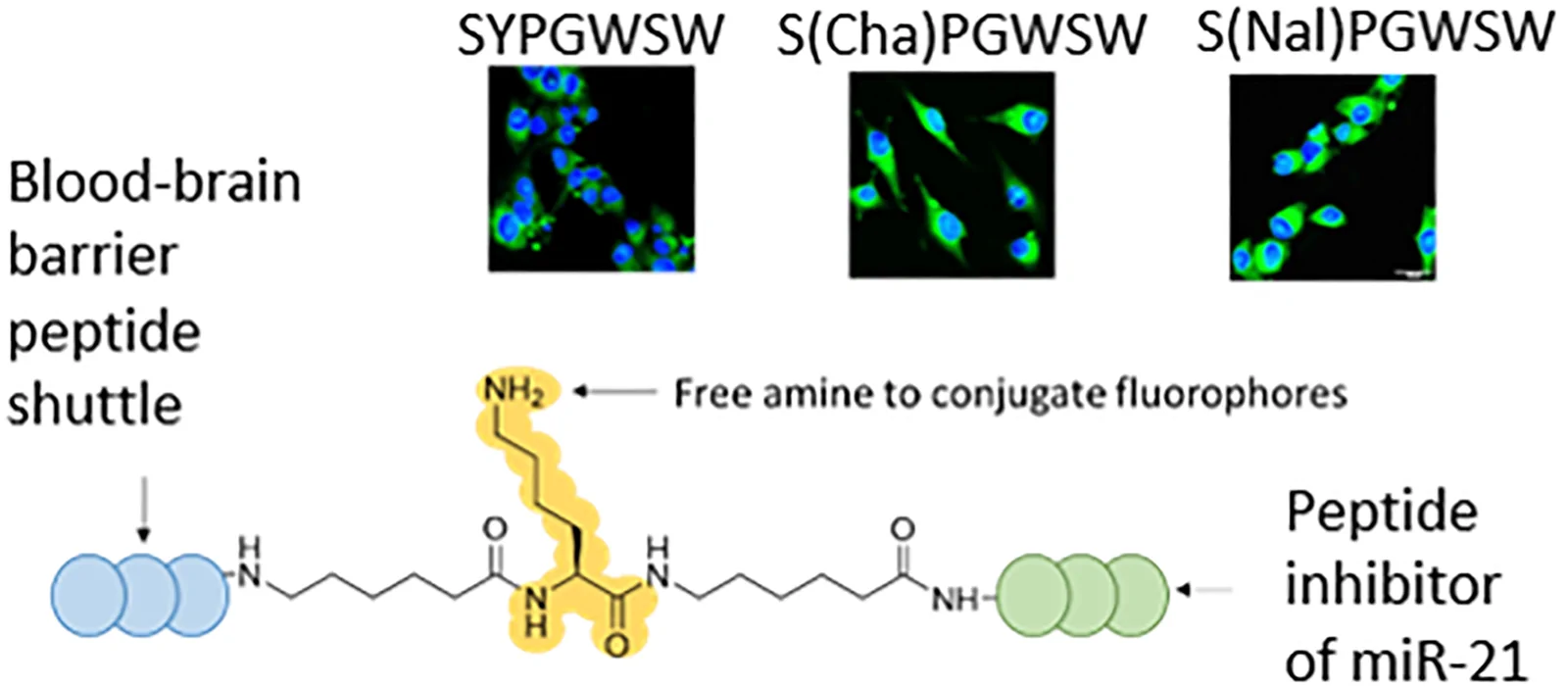

## Introduction

Gliomas are a heterogenous group of central nervous system (CNS) tumours characterised by distinct histological, genetic and epigenetic features.^1^ The outcomes for the most severe form, glioblastoma (GBM), remain dire, with an estimated 5-year survival rate of just 6.9%.^1^ This largely reflects the inability of the standard interventions such as surgical resection, temozolomide and radiotherapy to eliminate the disease. Further, despite their remarkable success in some cancers, checkpoint inhibitors such as nivolumab have shown limited benefit in GBM.^2^ Therefore, novel targeted GBM therapies are urgently required to improve patient outcomes.

MicroRNA-21 (miR-21-5p) is elevated in most solid tumours, drives multiple elements of carcinogenesis and is consistently elevated in grade III/IV GBM.^3^ The roles of miR-21-5p in GBM were first reported by Kosik and co-workers, who showed knockdown of miR-21 in cultured GBM cells triggered caspase activation, increasing apoptotic cell death.^4^ Subsequently, miR-21-5p was found to behave as an anti-apoptotic factor in GBM through acting on a plethora of targets.^5–9^

Peptides have emerged as a novel approach for the development of therapeutics for miRNA inhibition.^10^ These peptides typically function by inhibiting the processing of either primary miRNA (pri-miRNA) or precursor miRNA (pre-miRNA) rather than inhibiting the direct function of mature miRNAs the way anti-miRNAs do. Several peptide inhibitors of miRNA processing have so far been discovered mainly *via* high-throughput screening methods like microarrays,^11,12^ electrophoretic mobility shift assays,^13,14^ catalytic enzyme-linked click chemistry assay,^15^ and phage display.^16,17^ As highlighted in our recent review,^10^ these efforts have yielded 9 peptides and peptidomimetics that bind double stranded RNA junctions or grooves near the hairpin loop of pri-miRNAs or pre-miRNAs to inhibit biogenesis of mature miRNA. Unsurprisingly, the focus has been on inhibition of oncogenic miRNA, with 5 of the 9 peptides targeting pri-miR-21 or pre-miR-21,^12,13,15–17^ two targeting pre-miR-155,^11,14^ and one targeting pre-miR-23b,^18^ leaving only early work on *C. elegans* pre-miR-39 as the case where the target was not explicitly linked to cancer.^19^

The blood brain barrier (BBB) helps maintain normal brain function but also prevents systemic delivery of therapies to the brain, which is a major hurdle for GBM treatment.^20^ As a result, cell penetrating peptides (CPPs) with BBB-penetration capabilities have been investigated extensively as a tractable approach for delivering drug payloads across the BBB, and were recently collated in the B3Pdb database.^21^ The quorum-sensing SYPGWSW peptide (1) is one such BBBpS,^22^ as is its retro-inverso d-amino acid counterpart.^23^ These short lipophilic peptides exhibited exceptional tumour homing properties in an intracranial U87 glioma mouse model.^23^ The peptide was internalised by brain capillary endothelial cells, human umbilical vein endothelial cells (HUVEC) and U87 GBM cells.^23^ Further, mechanistic studies showed that the peptide primarily entered cells *via* energy-dependent caveolae mediated endocytosis.^23^

This study aimed to understand the properties of 1 that facilitated cellular uptake in GBM. We show that the aromatic residues are central to function while the Pro is not essential. Importantly, substitution of Tyr and, to a lesser extent, Trp with unnatural cyclic amino acid residues enhanced penetration of the peptides into GBM cells, with the aromatic 1-naphthylalanine substitutions being more effective than the aliphatic cyclohexylalanine (Cha). In addition, we evaluated a tandem fusion peptide, in which 1 was conjugated to ALWPPNLHAWVP, a peptide previously reported to inhibit miR-21 processing into the mature functional form.^16^

## Results and discussion

### Identification of functionally important residues in a BBB peptide shuttle

Peptides were synthesised on a Rink Amide linker resin using standard Fmoc/*t*Bu solid-phase peptide synthesis (SPPS) with an N-terminal 6-aminohexanoic acid (Ahx) linker to enable N-terminal labelling with FITC to visualise peptide uptake and distribution in GBM cells (Table 1). To define their contributions to the cell penetrating ability of 1, aromatic amino acids and proline were sequentially substituted with Ala. Uptake of 3, 5 and 6 was decreased by 50%, 85% and 64%, respectively, compared to 1, likely due to their reduced lipophilicity, as indicted by lower calculated partition coefficient (cLog *P*) values generated by Chemicalize (ChemAxon) software (Fig. 1A and B and Table 1). In contrast, changing Pro to Ala (4) had no significant effect on peptide uptake. These observations suggest that the Tyr and Trp aromatic residues of 1 were important for peptide uptake, while the imino acid Pro seemed less crucial.

**Table 1:** Sequences of peptides investigated in this study

Peptide	Sequence	cLog *P*	*S* Value
1	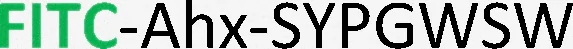	2.70	1.2
2	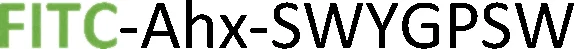	2.70	2.1
3	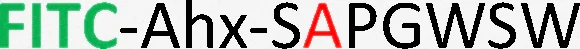	1.35	6.0
4	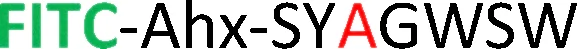	n.d.	1.7
5	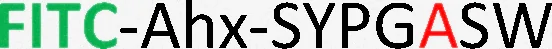	0.95	2.6
6	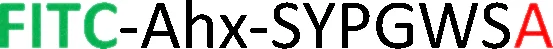	0.95	2.5
7	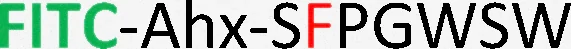	3.01	4.6
8	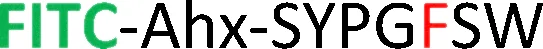	2.60	1.6
9	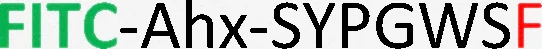	2.60	1.6
10	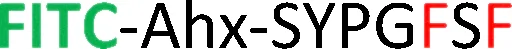	2.50	1.6
11	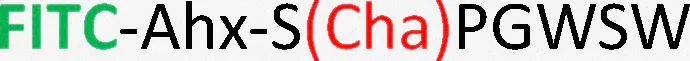	3.47	n.d.
12	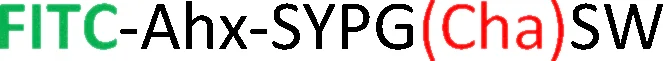	3.07	n.d.
13	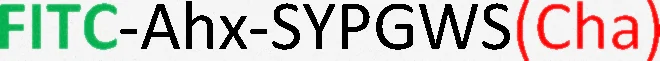	3.07	n.d.
14	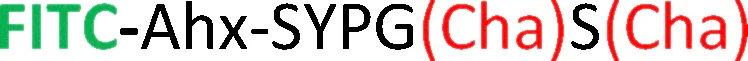	3.44	n.d.
15	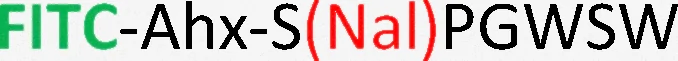	3.99	n.d.
16	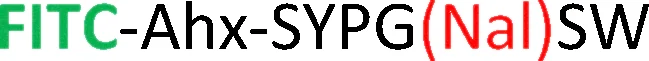	3.59	n.d.
17	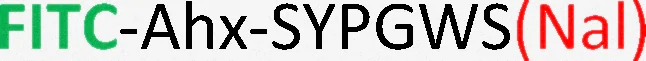	3.59	n.d.
18	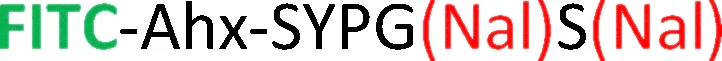	4.48	n.d.
19	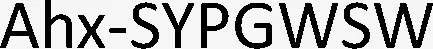	n.d.	1.1
20	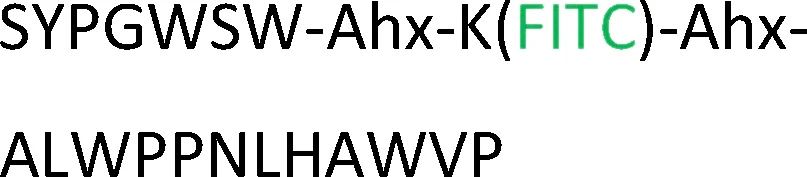	n.d.	2.1
21	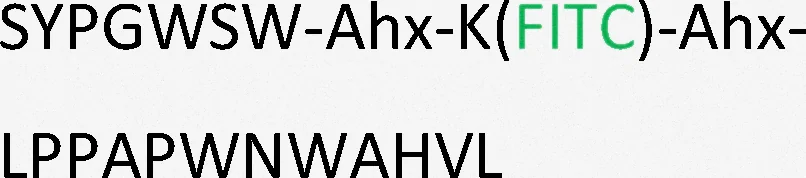	n.d.	4.4
22	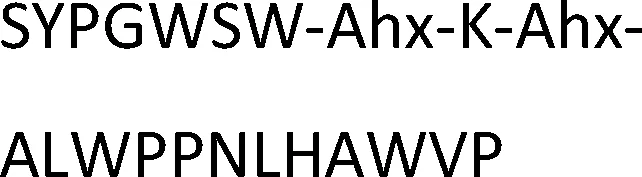	n.d.	2.1
23	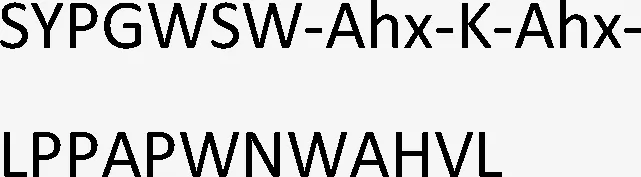	n.d.	3.1

**Fig. 1:**
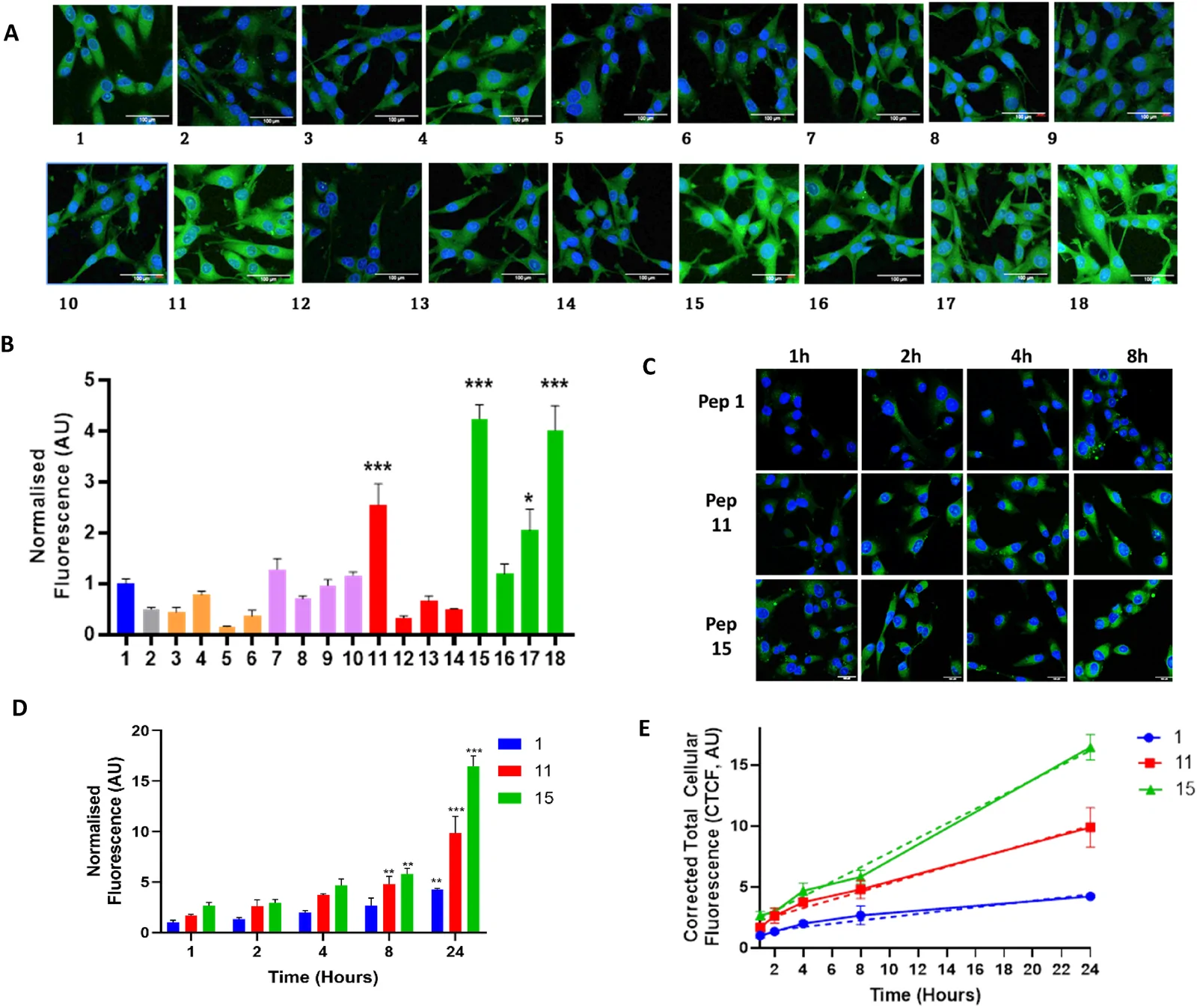
Uptake of 1 and variants thereof in U87 cells. (A) Confocal micrographs of cells treated with 10 µM of indicated FITC-labelled peptides (green) for 24 h at 37 °C. Nuclei were stained with DAPI (blue). (B) Quantitative analysis of peptide uptake in U87 cells. Corrected total cell fluorescence was normalised to peptide 1. (C) and (D) Time-course of peptide uptake as revealed by confocal microscopy (C), followed by quantification (D), which includes the 24 h timepoint. (E) Line plot (solid lines) and linear regression curves (dotted lines) for estimation of the rate of peptide uptake over 24 h. Error bars represent SEM; statistical analysis was one-way ANOVA with Dunnett's multiple comparisons test compared to peptide 1. *P < 0.05, **P < 0.01, ***P < 0.001. Scale bar: 100 µm.

We next determined whether an alternative aromatic amino acid, Phe, was tolerated in place of Tyr or Trp. There was no significant difference in the uptake of 7, 8, 9 and 10 compared to 1 (Fig. 1A and B). Thus, while small Ala impairs cell penetration, bulky hydrophobic Phe can substitute for Tyr and Trp in driving internalisation of the peptide.

We recently established molecular determinants that distinguish BBBpS from CPPs more broadly.^24^ This methodology ranks peptides based on specific physicochemical properties such as size, charge, aromaticity and hydrophobicity, generating *S* values for brain targeting. Typically, good translocators have *S* < 1.5, and poor translocators have *S* > 10. We determined *S* values for a selection of peptides in Table 1 and, gratifyingly, observed that 1 had a score of 1.2 compared to an *S* value of 2.1 for the scrambled control 2. Substituting Trp for Ala or Phe had the most dramatic effect on the *S* value, raising it to 6.0 or 4.6, respectively, whereas replacing Pro or Trp with Ala had more modest effects. Thus, the Tyr residue appears to be a key determinant of the BBBpS function of 1.

### Effects of lipophilic amino acid substitutions

Given that Phe substitutions did not affect cell penetration by 1, we were intrigued as to whether peptide uptake was mainly related to the lipophilic nature of Tyr, Trp and Phe residues or perhaps associated with their ability to form π interactions or cation-π interactions with membrane components. To shed light on the putative roles of lipophilicity, Tyr and Trp were substituted with cyclohexylalanine (Cha), which has a non-aromatic yet lipophilic ring.

Strikingly, a significant 2.5-fold increase in the uptake of 11 was observed, for the peptide with Tyr substituted with Cha (Fig. 1 A,B; Fig. 1). Since Cha lacks the π electron cloud of Phe and primarily interacts through van der Waals interactions, this suggest that substituting Tyr at position two with a non-aromatic lipophilic residue likely enhances uptake through hydrophobic mechanisms. In contrast, substitutions of Trp with Cha, either individually, as in 12 and 13, or together, as 14, had little or no effect on peptide uptake, even though 14 in particular was as lipophilic as 11.

Hence, replacement of Tyr, but not Trp, appears to be a key mechanism for improving the function of the peptide.

Taken together, the above observations suggest that replacement of Tyr but not Trp residues with Cha promotes internalisation of the L-WSW peptide. To further investigate the importance of the aromatic side chain, the effects of substituting Tyr and Trp with 1-naphthylalanine (1-Nal) were investigated, given that this unnatural amino acid has a greater propensity to participate in π interactions and is structurally similar to Trp.^25,26^ Substituting Tyr with 1-Nal raised the internalisation of 15 by 4-fold compared to 1 (Fig. 1A and B). Likewise, replacement of Trp with 1-Nal in 17 but not 16, improved uptake, as did changing both Trp residues to 1-Nal in 18, which boosted peptide accumulation in cells by 4-fold relative to 1.

Indeed, the true increase in levels of internalisation of 15 and 18 compared to 1 was probably greater than 4-fold, as the fluorescent signal was typically saturated for cells exposed to 15 and 18, a consequence of deploying the same image acquisition parameters throughout the study.

Interestingly, although 11, 14, 16 and 17 had comparable levels of predicted lipophilicity, only 11 was taken up more significantly than the less lipophilic parent peptide 1. This suggests increased lipophilicity is not sufficient to enhance uptake. Nonetheless, as the two peptides with the highest levels of uptake, 15 and 18, were also the most lipophilic according to cLog *P* predictions, elevated lipophilicity may cooperate with increased π–π stacking between peptide and membrane protein aromatic residues to promote uptake of the peptides. That said, 11 and 14 have similar predicted lipophilicities but the uptake of 14 was dramatically reduced compared to 11. This may be due to impaired interactions between 14 and membrane-associated glycosaminoglycans (GAGs), since Cha substitutions in 14 remove the Trp side chain π system implicated in GAG interactions.^27^ Such π system loss could also contribute to the impaired uptake of 12 and 13. Alternatively, there may be specific molecular recognition or membrane partitioning properties that are lost when Trp is replaced with Cha. In addition, 11 and 15 were studied further in the unrelated U251 GBM cell line to confirm the enhanced internalisation was applicable to other GBM cell types. As with U87, elevated uptake of 11 and 15 was observed in U251, with both Cha and 1-Nal substitutions driving a 4-fold increase in levels accumulated in the cells after 24 h (Fig. S1).

### Studies on the time-course of peptide uptake

Early studies suggested CPP uptake peaks between 1 and 3 h after application to cells.^28^ Therefore, the levels of peptide accumulation in U87 cells was monitored at 1–8 h post treatment and compared to the earlier 24 h data. Only limited fluorescence was observed at shorter time points, reaching approximately 2, 4 and 5-fold for 1, 11 and 15 at 4 h (all relative to peptide 1 at 1 h) compared to 4, 10 and 16-fold at 24 h (Fig. 1C and D).

To more formally estimate the differences in the rates of peptide uptake, fluorescent intensities were plotted against time and the gradients of the curves determined (Fig. 1E). The gradients of the curves for 1, 11 and 15 were 0.13. 0.34 and 0.60, respectively. Taken together, these observations indicated that Cha and 1-Nal modifications facilitate accelerated internalisation of 11 and 15 in U87 cells compared to 1.

### Mechanism of peptide cellular internalisation

Earlier studies had revealed that 1 penetrated U87 cells *via* energy-dependent endocytosis and lipid rafts.^23^ Thus, the mechanism of uptake for peptides 11 and 15 was examined, as the modification may have altered uptake mechanisms. The internalisation of the peptides was significantly hindered following treatment with NaN_3_, indicating the transport of all three peptides into the cells followed an energy-dependent pathway (Fig. 2A and Fig. S2A). Likewise, treatment with the lipid raft-mediated endocytosis inhibitor MβCD also impaired uptake, indicating that peptide internalisation followed the lipid-raft pathway to endosomes (Fig. 2A and Fig. S2A). Localisation of 1, 11 and 15 to endosomes was verified by labelling endosomes with Lysotracker Red DND-99, and co-localisation between the peptides and the endosomal marker was observed in the perinuclear region (Fig. 2B). Therefore, these observations indicate that the Cha and 1-Nal modifications of 11 and 15 did not affect the mechanisms of uptake. However, these findings do not exclude the possibility that other transport mechanisms may be involved in penetration of the peptides into the U87 cells, particularly macropinocytosis. Therefore, additional studies using specific inhibitors of these alternative pathways are required to further enhance our understanding of how the BBBpS peptides are conveyed into GBM cells.

**Fig. 2:**
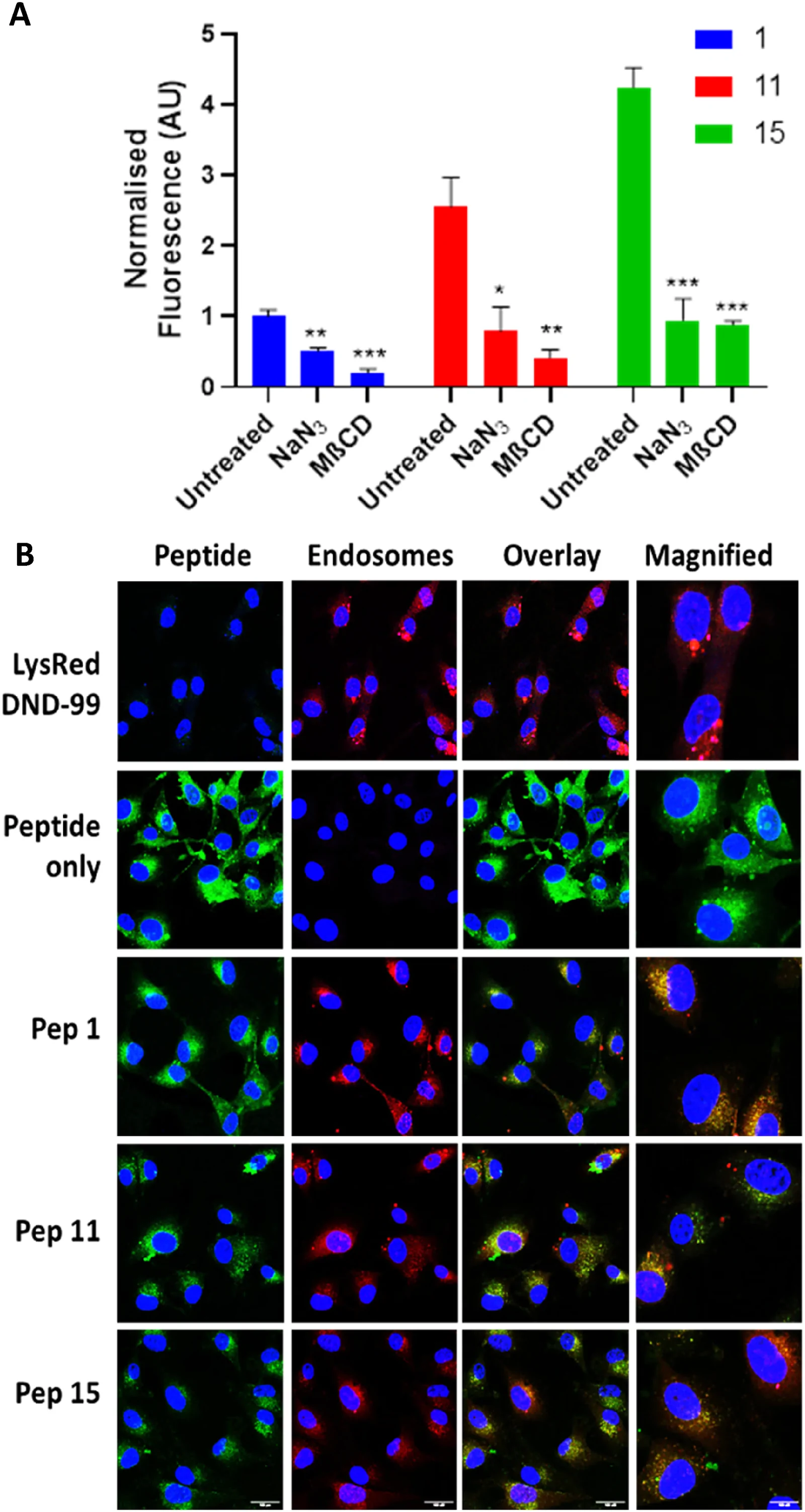
Mechanism of Peptide Cellular Internalisation in U87 cells. (A) Peptide uptake in the presence of inhibitors. Cells were pre-incubated for 1 hour with NaN_3_ (10 mM) or MβCD for (5 mM), respectively. Subsequently, FITC-labelled peptides were applied to cells at 10 µM for 24 h. Peptide fluorescence was quantified across 30 cells from 5 fields of view. Error bars represent SEM (*n* = 3). (B) Co-localisation of peptides with the endolysosomal pathway was observed using FITC-labelled peptides (green) applied to cells at 10 µM for 24 h followed by 1 h treatment with Lysotracker Red (LysRed DND-99; red). Nuclei were stained with DAPI (blue). Scale bar: 100 µm. Statistical analysis carried out using one-way ANOVA with Dunnett's multiple comparisons test. Values from treated cells were compared to untreated in each case; *P < 0.05, **P < 0.01, ***P < 0.001; ns, not significant.

### Lipophilic modifications enhance serum stability of the peptides

Next, we wanted to confirm that the different cellular uptake levels were associated with the new amino acid properties and not differences in the stability of the peptide. For these studies, peptides were incubated in 25% v/v human serum diluted in phosphate-buffered saline (PBS). After incubation, serum proteins were removed by precipitation prior to HPLC analysis. Initially, trichloroacetic acid (TCA; 7 to 15% in H_2_O) was employed to precipitate the serum proteins. However, due to the lipophilic nature of the peptides, quenching of serum proteins with TCA also precipitated the peptides from the reaction mixture. Organic solvents including ethanol, acetone, acetonitrile and methanol were explored as alternatives. The serum proteins precipitated in methanol and acetone but not in ethanol or acetonitrile (data not shown). Thus, methanol was employed to precipitate serum proteins at the end of each stability study time point.

The stability of the 1, 11 and 15 in 25% human serum was determined at specific time points over 24 h. The peptides were relatively stable to proteolysis, since over 70% remained intact following 8 h in 25% human serum (Fig. 3A). However, after 24 h in serum, peptide 1 and 11 had degraded significantly, with only 39% and 31%, respectively, of intact peptide remaining, whereas 15 was still 61% intact (Fig. 3B). This indicated that peptide 15 was significantly more resistant to proteolytic degradation than 1 and 11.

**Fig. 3:**
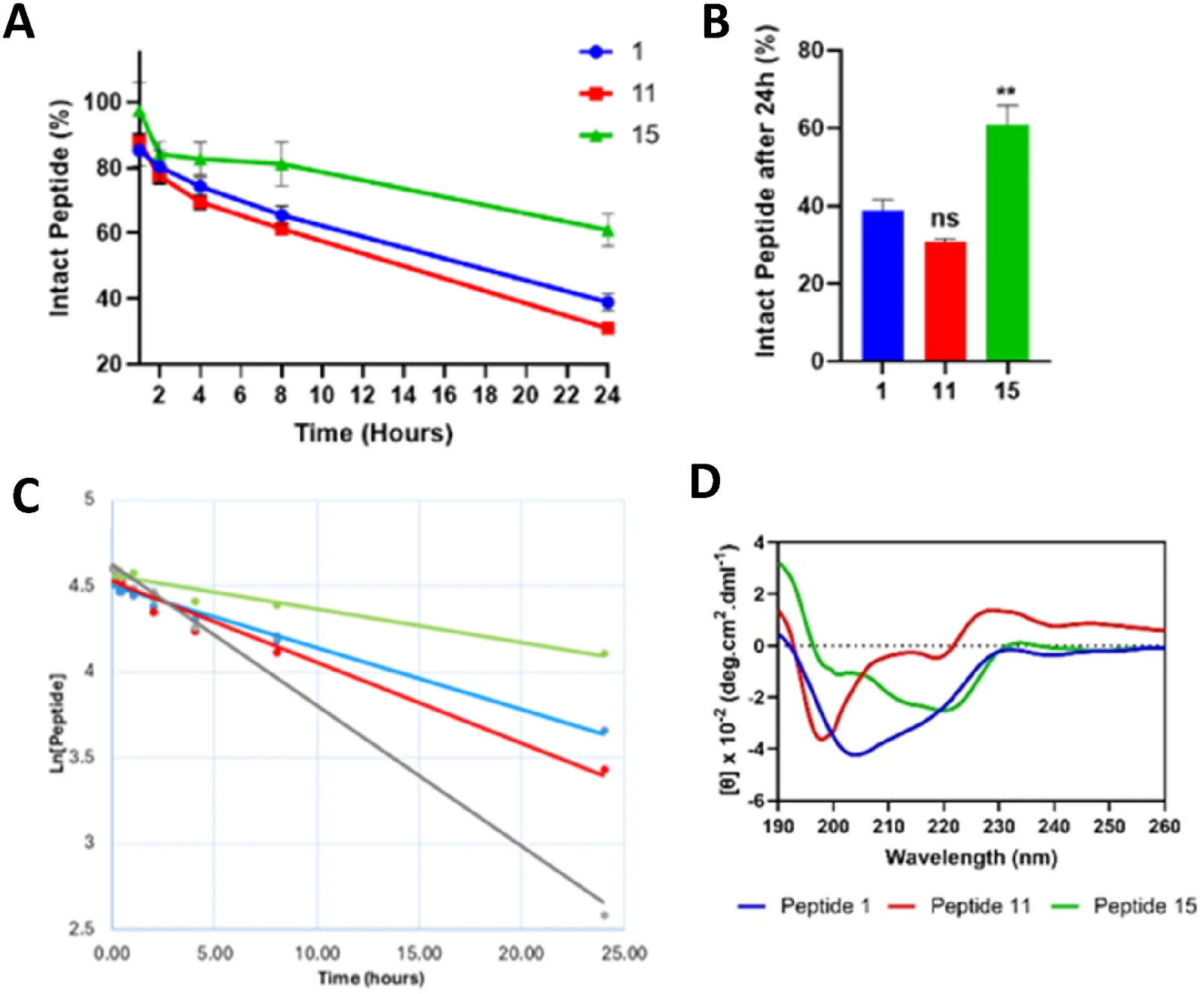
Proteolytic stability of 1, 11 and 15. (A) Time course following peptide stability in human serum (25% v/v) over 24 h. (B) Degradation of 11 and 15 relative to 1 after 24 h of exposure to human serum. (C) Graph employed to determine the half-lives of peptides 1 (blue), 11 (red), 15 (green) and 19 (black). (D) The secondary structure of 1, 11 and 15 as revealed by circular dichroism (CD). The CD spectra were obtained following 10 consecutive scans per sample (10 µM) after subtraction of the buffer (distilled water) baselines. (D) ****Error bars represent SEM; statistical analysis carried out using one-way ANOVA with Dunnett's multiple comparisons test: levels of significance **P < 0.01; ns, not significant.

Given the presence of a FITC label on the N-terminal amine of the peptides, we hypothesized that the stability of the peptides over the 8 h time frame may be associated with FITC-dependent protection from aminopeptidase-mediated proteolysis. To investigate this further, the stability of 1 in human serum was compared to the unlabeled counterpart 19 with a free N-terminal amine. Surprisingly, the stabilities of 1 and 19 were comparable over the first 8 h of serum exposure but diverged over 24 h, with peptide 19 significantly degraded to leave 13.3 ± 1.7% intact peptide at 24 h, compared to 39.0 ± 2.6% for peptide 1 (Fig. S3A and B). Thus, the conjugation of FITC at the *N*-terminus of the peptides likely increased their stability, probably by binding serum proteins and limiting access to proteolytic enzymes.

The half-lives of the peptides were determined by plotting the natural logarithm of peptide concentration (ln[*A*]) against incubation time. The degradation profile for each peptide followed apparent first-order reaction kinetics, as indicated by the *R*^2^ > 0.9 in the ln[*A*] *vs.* time graph (Fig. 3C). The rate constant (*k*_elm_) was obtained from the gradient of the ln[*A*] *vs.* time graph after linear regression fitting and used to determine the half-lives of the peptides from first order of reaction equation. The half-life of 15 was almost double that of 1 (∼35 h for 15 compared to ∼19 h for 1). In contrast, the half-life of 11 (∼15 h) was reduced compared to 1 (Table 2). Together, these observations indicate that the replacement of Tyr with Nal is a potent modification for enhancing the proteolytic stability of the peptide.

**Table 2:** Peptides half-lives in diluted human serum

Peptide	Equation	*R* ^2^	*k*	*t*_½_ (h)	%HSA-binding
1	*y* = −0.036*x* + 4.5035	0.97	0.04	19.25	64 ± 0.7%
11	*y* = −0.047*x* + 4.527	0.97	0.05	14.74	73 ± 0.6%
15	*y* = −0.0195*x* + 4.560	0.91	0.02	35.54	73 ± 0.2%
19	*y* = −0.0817*x* + 4.623	0.98	0.08	8.48	n.d.

To determine whether the enhanced stability of 15 was associated with secondary structural changes, the peptides were analysed by circular dichroism. The spectral signatures (Fig. 3D) of all three peptides were characteristic of the random-coil conformation, perhaps with some α-helical elements in 1. This indicates that the modifications in 11 and 15 did not stabilise secondary structure and the higher stability of 15 cannot thus be linked to structural changes.

We next sought to determine whether the enhanced stability of 11 and 15 was associated with increased binding to human serum albumin (HSA), which is known to help protect lipid-tagged peptides.^29^ Both 11 and 15 appeared to bind marginally more strongly to HSA than 1 (Table 2). However, there was no difference between the binding of 11 and 15 to HSA, despite the >2-fold increase in the stability of 15 compared to 11. Thus, although naphthyl alanine analogues have been reported to bind HSA,^30^ the resistance of 15 to degradation is unlikely to be due to a protective effect associated with HSA binding, but rather is more likely explained by resistance to serum endopeptidases. The mechanistic basis for such resistance to proteolytic degradation warrants further investigation but could include steric effects or reduced protease recognition associated with the 1-Nal residue. Indeed, the higher cLog *P* of 15 compared to 1 and 11 (Table 1) raises the possibility that there is self-assembly of 15 that may contribute to resistance against proteolytic degradation.

### Evaluation of BBB translocation and integrity *in vitro*

The effect of the Cha and Nal substitutions on the ability of the peptides to traverse the BBB was evaluated using a previously optimised *in vitro* model of the BBB. The model consists of human cerebral microvascular endothelial cells (HBEC-5i) on transparent polyester (PET) membrane cell culture inserts as described elsewhere.^31^ Fluorometric analysis confirmed spectral properties of the FITC-labelled peptides were comparable in terms of excitation and emission spectra and limits of detection in both PBS and media (data not shown). Initial toxicity testing confirmed no loss of viability upon 24 h exposure of HBEC-5i to the peptides at a concentration from 0.2–100 µM (Fig. S4). Next, peptides were applied to the apical side of the HBEC-5i monolayers, using three different peptide concentrations to enable putative dose–response relationships to be uncovered. Media samples were collected from both the apical and basolateral sides of the transwell culture inserts.

Limited translocation from the apical to the basolateral chamber was observed 1 h after application of the peptides (Fig. 4). For the scrambled control peptide 2, only ∼20% and ∼34% had entered the basolateral fluid after 8 and 24 h, respectively. In contrast, ∼52%, 44% and 48% of peptides 1, 11 and 15, respectively, had crossed the barrier following 8 h of exposure to 5 µM or 10 µM (Fig. 4). After 24 h of exposure, the proportion of 1 and 15 detected in the basolateral chamber had increased further, reaching ∼60%, while for 11, the proportion translocated was ∼50%. Notably, raising the peptide concentration to 25 µM did not lead to a proportional increase in permeation across the BBB and indeed tended to decrease uptake, conceivably due to saturation of putative receptors or concentration-dependent phenomena such as aggregation. These findings indicate that the Nal substitution in 15 did not impede the ability of the BBBpS to translocate across the BBB, while the Cha modification was associated with a small but significant drop in translocation efficiency (Fig. 4E). In general, the increase in BBBpS intensity in basolateral media over time was mirrored by a concomitant reduction in the amount of peptide detected in the apical chamber. This suggests that the Cha but not the Nal substitution may slow trafficking of the BBBpS through the endothelial monolayer of the BBB.

**Fig. 4:**
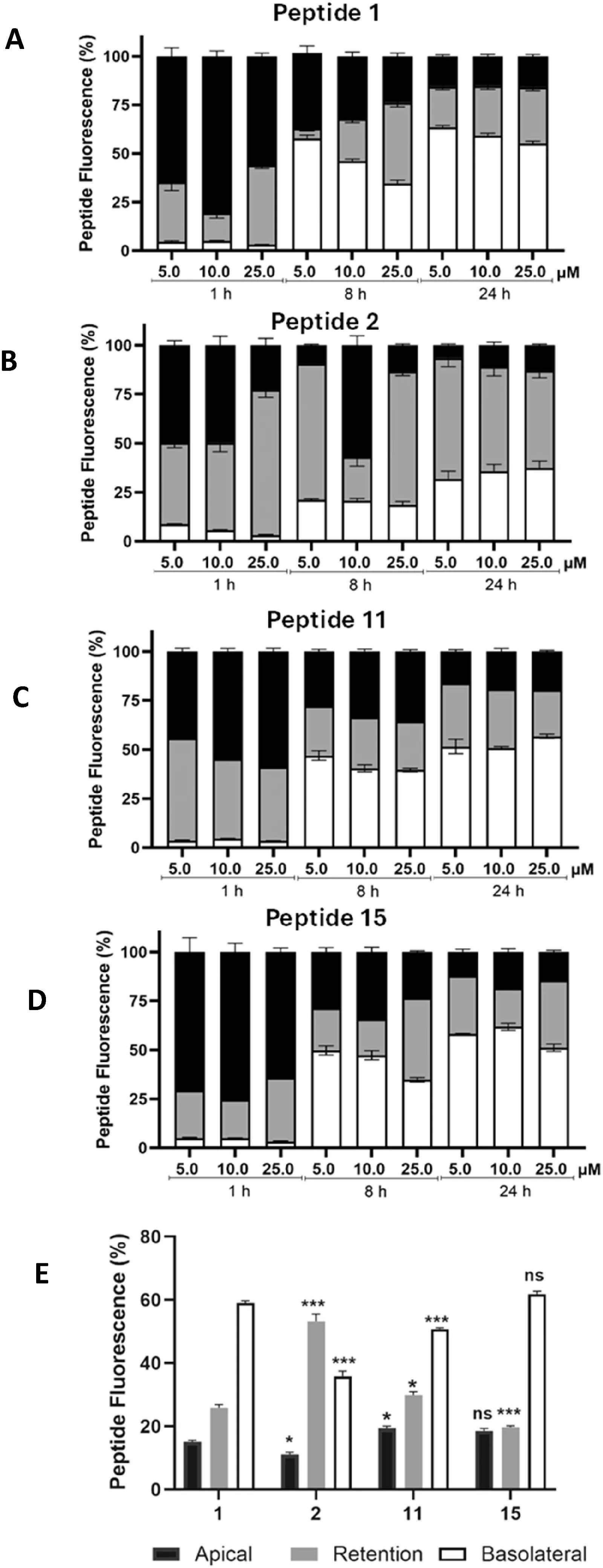
Peptide translocation across the blood–brain barrier (BBB). For A–D, the indicated peptides were added to the apical side of the *in vitro* BBB model at 5, 10 or 25 µM and incubated for the indicated time points. The amounts of peptide in the apical and basolateral chambers and retained in the HBEC-5i cells were measured. All peptides were labelled with FITC for fluorescent detection. Experiments were performed in triplicates. (E) Comparison of the 10 µM data at 24 h. Error bars represent SEM. Statistical analysis carried out using two-way ANOVA with Dunnett's multiple comparisons test: levels of significance *P < 0.05, **P < 0.01, ***P < 0.001; ns, not significant. Apical, retention and basolateral data for all the peptides were compared to those of 1.

### Silencing miR-21-5p with a fusion peptide

Finally, to test whether it was possible to silence miR-21 in GBM cells, we generated a peptide conjugate 20 consisting of 1 in tandem fusion to a peptide that Bose and colleagues previously identified as an inhibitor of pre-miR-21 processing to mature miR-21.^16^ An inert spacer sequence consisting of 6-amino hexanoic acid (Ahx) and lysine (Ahx-Lys-Ahx) was included to separate the distinct peptide functionalities whilst the lysine residue also provided an anchor for fluorescent labelling to investigate the localisation of the peptide conjugate. First, we assessed the ability of 20 to penetrate U87 cells. The localisation of 20 was comparable to that of 1, and quantitative analysis indicated that cellular uptake of the peptides was comparable (Fig. 5A). Hence, the cell penetrating function of 1 was retained following conjugation to a bioactive peptide. Next, the ability of 20 to translocate across the BBB was examined. Toxicity studies indicated that none of the peptides tested impaired the viability of brain endothelial cells or the integrity of the BBB (Fig. S4). Subsequently, the ability of 20 to transverse the BBB was determined with the *in vitro* BBB model. Following 1 h of incubation, 20 had translocated the model BBB by 3%, while after 24 h of incubation, the amount of translocated peptide had risen to 45% (data not shown). Notably, the efficiency of translocation of 20 across the BBB model into the basolateral chamber was slightly lower than that of 1, and that of scrambled control peptide 21 was lower still (Fig. 5B). Higher levels of 20 and 21 were observed in both the apical chamber and the retention (brain endothelial cell) layer, suggesting both entry into and migration across the BBB was slowed by the physicochemical differences of 20 and 21 compared to 1. These differences were unlikely to be associated with conformational changes, as circular dichroism spectra were similar (Fig. S4). These data demonstrated the ability of peptide 1 to deliver peptide cargo across a model of the BBB. Finally, to evaluate the ability of the fusion peptide to suppress miR-21 expression, U87 cells were treated with the peptide conjugate 22, which is identical to 20 but lacks the FITC label. Treatment of U87 cells with peptide 22 for 24 h led to a 15-fold reduction in miR-21 expression relative to scrambled control peptide conjugate 23 (Fig. 5C). These data suggest a functional interaction between 22 and its pre-miR-21 target. However, the trafficking events connecting BBB translocation, cellular internalization, intracellular trafficking, and target engagement were not directly examined in this study. Consequently, the mechanism leading to miR-21 suppression remains inferred from the biological response that suggests interaction between 22 and its pre-miR-21 target. It would be interesting to explore the impact of endosomal acidification and maturation inhibitors on the ability of 22 to downregulate miR-21-5p, in order to confirm that endosomal escape is a prerequisite for function. Further studies are also required to demonstrate direct binding of 22 to pre-miR-21 in the intracellular context, for example *via* complementary peptide and RNA pulldown assays or Förster resonance energy transfer (FRET) studies.

**Fig. 5:**
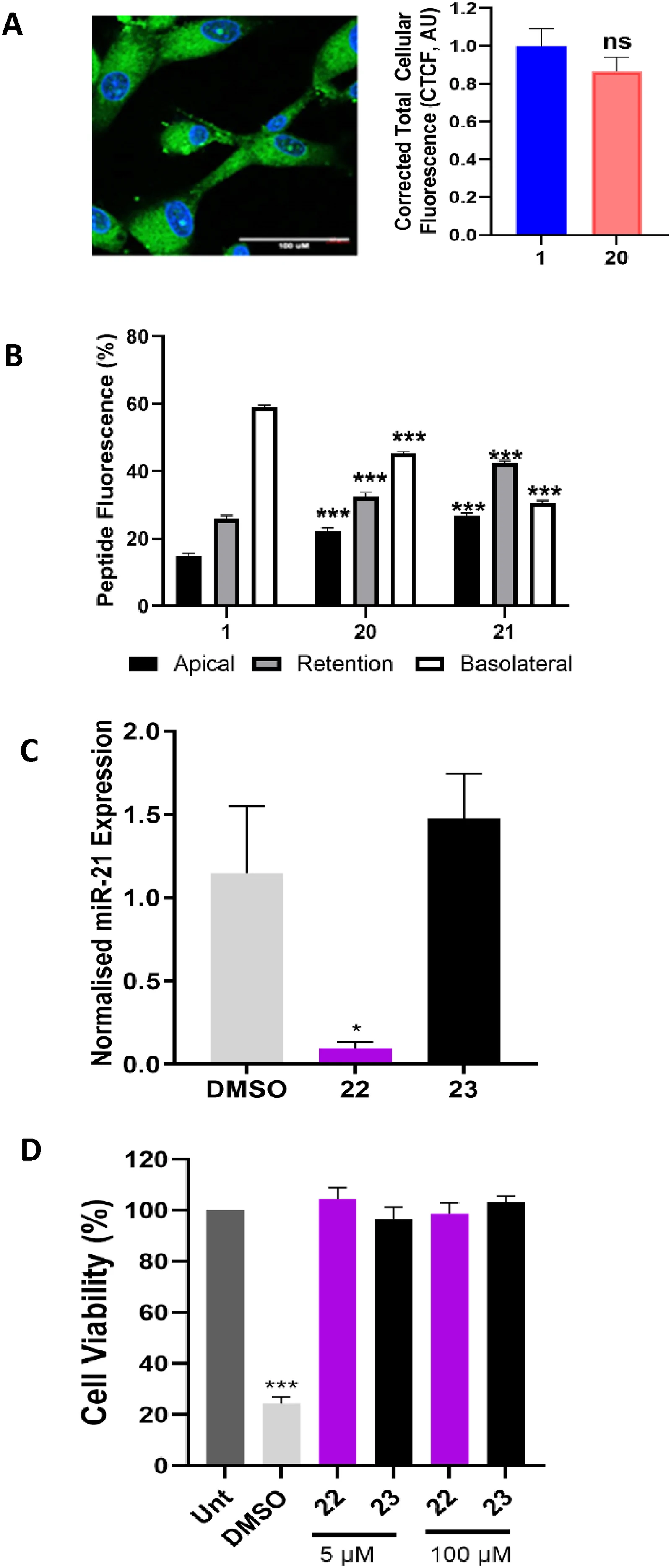
Evaluation of peptide conjugates in U87 cells. (A) Confocal micrographs of U87 cells treated with 10 µM of 20 for 24 h. Nuclei stained with DAPI (blue). Graph depicts the quantitative fluorescence from cells loaded with 20 normalised to those loaded with 1. (B) Translocation of 20 across the BBB at 24 h, compared to data for 1 from Fig. 4E. (C) Silencing of miR-21-5p by unlabelled peptide 22 compared to scrambled control 23. Cells were treated with 10 µM of peptide in complete medium for 24 h prior to analysis. (D) Analysis of U87 cell viability after exposure to 22 or 23 for 24 h was performed using the Alamar Blue assay. Unt, untreated. Positive control 40% DMSO. Error bars represent SEM from 3 independent experiments; statistical analysis was carried out using unpaired *t* test with Welch's correction for (A), two-way ANOVA with Dunnett's multiple comparison test for (B) and one-way ANOVA with Dunnett's multiple comparisons test for (C) and (D). **P < 0.05, ***P < 0.0001. Scale bar = 100 µm.

Interestingly, there was no significant change in the viability of cells exposed to 22 compared to 23 (Fig. 5D) Together, these findings indicate that 22 silences miR-21 robustly in GBM cells but this is not sufficient to promote cell death by itself. This is consistent with early studies linking the value of miR-21-5p silencing to sensitisation of GBM cells to radiotherapy and chemotherapy rather than direct reduction of cell viability.^32–34^ Therefore, further studies are warranted to test the ability of 22 enhance the efficacy of chemotherapeutic and radiotherapeutic interventions in GBM, along with analyses of miR-21-5p target expression to reveal which ones are modulated most in the presence of 22. In addition, although we generated 22 because 1 had been established as a BBBpS, it will be interesting to redesign 22 with the BBBpS portion replaced with 15 given the enhanced stability conferred by 1-Nal.

## Conclusions

In this study, we demonstrated the functional silencing of an oncogenic miRNA using a peptide fusion comprising a BBBpS and a peptide that inhibits miRNA processing. Numerous peptides have been shown to cross the BBB^21^ and several peptides that inhibit miR-21 maturation have been reported.^10^ Our findings showcase the potential of a single fusion peptide that crosses the BBB and silences miRNA. Further studies are required to exploit the stabilised BBBpS 15 in the context of such a fusion peptide and it would be interesting to evaluate a d-isomer version of 15, as has been reported for 1.^23^ Notably, previous reports on 1 have demonstrated transport across BBB models and brain accumulation *in vivo*^22,23^ yet without clarification of the underlying transport mechanism. The non-linear concentration dependence observed here is therefore compatible with a saturable transport process and may suggest the involvement of receptor-assisted transcytosis, although additional mechanistic studies will be required to distinguish this possibility from alternative explanations, including concentration-dependent self-association or altered membrane interactions. In addition, the current work does not demonstrate the endolysosomal compartment localization of 22 and subsequent escape to the cytosol for access to the pre-miR-21 target. Hence, further mechanistic studies are required to define the relative endosomal:cytosolic levels of 22. Nonetheless, the relative simplicity with which a tandem peptide fusion can be generated compared to anti-miRNA oligonucleotide conjugates to peptides or other targeting moieties may offer strategic advantages in the development of anti-miRNA therapeutics for GBM and other diseases.

## Experimental

### Materials

Fluorenylmethoxycarbonyl (Fmoc) N-protected amino acids were purchased from CEM Microwave Technology Ltd (UK), along with Rink Amide ProTide resin (100–200 mesh, 0.56 mmol g^−1^) and Oxyma Pure™. Fmoc N-protected 1-naphthyl alanine (Nal), cyclohexylalanine (Cha) and amino hexanoic acid (Ahx) were purchased from Glentham Life Sciences (UK). Acetic anhydride, acetonitrile, *N,N*-dimethylformamide (DMF), formic acid, methanol, palladium-tetrakis catalyst and trifluoroacetic acid were from Thermo Fisher Scientific (UK). *N,N*′-Diisopropylcarbodiimide (DIC), *N,N*-diisopropylethylamine (DIPEA), piperidine, trifluoroacetic acid (TFA), triisopropylsilane (TIPS), pyridine anhydrous (99.8%), ninhydrin, phenylsilane and fluorescein-5-isothiocyanate (FITC) were from Merck (UK). All reagents were HPLC or LC-MS/MS grade where relevant.

### Peptide synthesis

Peptides were prepared by automated microwave-assisted Fmoc solid-phase peptide synthesis (SPPS) methods using a Liberty Blue synthesiser (CEM Microwave Technology Ltd) using Rink amide ProTide resin (180 mg, 0.56 mmol g^−1^ loading; 0.1 mmol), with *N,N*′-diisopropylcarbodiimide (DIC; 1 M stock solution in DMF; 10 eq.), Oxyma Pure (1 M stock solution, 5 eq.) and piperidine (20% v/v in DMF; 587 eq., 4 mL) for activation, racemisation suppression and deprotection, respectively. Amino acids were double coupled at 90 °C for 2.5 min, with milder conditions (10 min, 50 °C) for thermally labile Fmoc-His(Trt)-OH. For peptides 1–18, a FITC label was attached on-resin to the N-terminal amine of an Ahx spacer in a solution comprising pyridine, DMF and DCM (12 : 7 : 5 v/v; 4 mL) shaking at room temperature overnight. A Kaiser test for free amines was performed to confirm completion of coupling. The resin was washed 3 times with DMF and diethyl ether, respectively, and cleaved from resin at the C-terminal amide using a cleavage cocktail comprising TFA, TIPS and water (8 : 1 : 1 v/v; 4 mL) with regular shaking for 4 h at room temperature. Cleaved peptide was precipitated by dropwise addition to cold diethyl ether, the suspension centrifuged (4000 rpm, 5 min) and the isolated pellet washed twice in diethyl ether. Peptides were then purified by preparative HPLC (Agilent Infinity 1260 equipped with a Waters XBridge® Peptide BEH C18 Prep 130 Å column) or flash chromatography on a Teledyne ISCO Combi*Flash®* NextGen 300+ instrument. Isolated pure peptides were concentrated *in vacuo* to the remove organic volatiles. and dissolved in water, flash frozen (liquid N_2_) and lyophilised. Adjustments made for synthesis of fusion peptides 20, 21, 22 and 23 are in the SI Methods. The HPLC and mass spectrometry traces for the purified peptides are in SI S2.

### Glioblastoma and BBB cell culture

The U87 and U251 cells were kind gifts from Amos Fatokun (Liverpool John Moores University) and Darius Widera (University of Reading), respectively. Cells were maintained in Dulbecco's modified Eagle's media (DMEM) supplemented with 10% newborn calf serum. All cell lines were cultured without antibiotics. Human cerebral microvascular endothelial cells (HBEC-5i, ATCC® CRL-3245^TM^) were cultured on 0.1% gelatin (Gibco/Thermo Fisher, USA)-coated T-flasks in DMEM:F12 medium (ThermoFisher, USA) as a monolayer. The medium was supplemented with 10% fetal bovine serum (FBS), 1% penicillin/streptomycin and 40 µg mL^−1^ endothelial growth supplement (ECGS) (Sigma-Aldrich, Spain), according to manufacturer's instructions.

### *In silico* predictive BBB-translocation

To calculate the theoretical capabilities of the peptides to cross the BBB, we applied a previously described predictive model.^24^ Initially, we calculated the physicochemical properties of each peptide. Secondly, we calculated the sum of squares (s), with lower S indicating greater capacity to cross.

### Cell-penetrating peptide uptake studies

For bioimaging, cells were seeded at 2 × 10^5^ cells on glass cover slips in 6-well plates or Ibidi glass bottom dish 35 mm (Thistle Scientific, UK) for 24 h. Fluorescently labelled peptide (10 µM) was applied to cells for indicated timepoints. To visualise, cells were washed with PBS, fixed using ice-cold methanol (MeOH) for 7 min at −20 °C and washed 3 times with PBS. Cells were mounted with 4′,6-diamidino-2-phenylindole (DAPI) containing Fluoromount G mounting medium (ThermoFisher Scientific, UK) and images acquired by confocal microscopy. To quantify the cellular uptake of FITC labelled peptides, images for each sample were captured from 5 different fields of view to span at least 30 cells. Fluorescence intensity was quantified using image J.

### Fluorometric analysis of peptides for BBB translocation studies

Spectroscopic data were recorded on a FS900 fluorometer (Edinburgh Instruments, UK). Peptides were dissolved at 100.0 µM in 1X PBS or medium. Spectra were recorded at room temperature. The limit of detection of each peptide was evaluated using the optimized excitation/emission wavelenghts (*λ*_exc_/*λ*_em_), which was *λ* 495/519. The peptides were diluted in a range between 0.05 and 100.0 µM in either 1X PBS or medium and the fluorescence measured using a Varioskan^TM^ LUX multimode microplate reader (Thermo Fisher, Spain).

### *In vitro* BBB translocation assay

The translocation capability of all peptides was assessed using an *in vitro* BBB model previously optimized using HBEC-5i cell line.^24,31^ Briefly, cells were allowed to grow until 80% confluence in a culture T-flask. Then, they were carefully harvested with trypsin-EDTA (Gibco/Thermo Fisher, USA) and seeded (8000 cells per well) into tissue culture inserts (transparent polyester (PET) membrane with 1.0 µm pores) for 24-well plates (BD Falcon, USA) pre-coated with attachment factor protein solution. The medium was changed every other day for 8 days, and, afterwards, cells were washed two times with 1x PBS and one time with DMEM:F12 without phenol red (Gibco/Thermo Fisher, USA). Next, peptides diluted in DMEM:12 without phenol red, at different concentrations (5.0, 10.0, and 25.0 µM), were added to the apical side of the *in vitro* BBB model and incubated for 1, 8, and 24 h. Finally, samples from the apical and basolateral side were collected.

Peptide translocation was determined by fluorescence intensity (*λ*_ex_ = 495 nm, *λ*_em_ = 519 nm), using a Varioskan^TM^ LUX multimode microplate reader. Samples recovery (%) were calculated as follows:
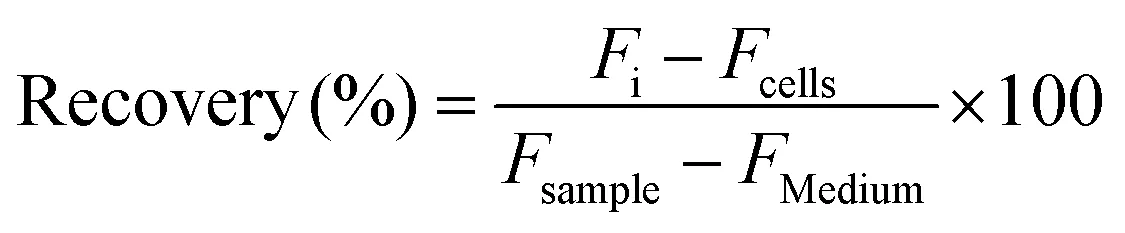
where, *F*_i_ is the fluorescence intensity of samples recovered, *F*_cells_ is the intrinsic fluorescence intensity of cells without samples incubation, *F*_sample_ is the intensity of total sample initially added to the transwell apical side, and *F*_Medium_ is the intensity of DMEM:12 medium without phenol red. Experiments were performed in triplicates on different days using three independently grown cell cultures.

### Human serum albumin binding by rapid equilibrium dialysis

Peptide binding to human serum albumin was estimated using rapid equilibrium dialysis (RED; ThermoFisher) according to the manufacturer's instructions. Human serum albumin (HSA) was dissolved with peptide solution (PBS, 10% DMSO) to yield a HSA/peptide mixture (400 mM HSA, 200 mM peptide). The HSA/peptide mixture (100 mL) was deposited into the red chamber of the RED device. Buffer consisting of PBS with 10% DMSO (350 mL) was added to the white chamber. The plate was sealed with parafilm and tape, covered in aluminium foil and shaken at 200 rpm for 24 h at 37 °C. The HSA/peptide sample (60 mL) from the red chamber was quenched with pure methanol (180 mL), placed on ice (30 min) then centrifuged at 13 200 rpm for 5 min. The resulting supernatant, along with the buffer sample from the white chamber, was assessed by analytical reversed-phase high-performance liquid chromatography (RP-HPLC) using an Agilent 1100 system equipped with a 100 mm Phenomenex Aeris peptide XB-C18 (3.6 mm particle size, 150 × 4.6 mm) column. A linear gradient of 20–60% acetonitrile (0.05% trifluoroacetic acid, TFA) *versus* water (0.05% TFA) over 10 min at 65 °C was used, with a flow rate of 1 mL min^−1^, pressure between 48–106 bar and UV detection at 214 nm.

The percentage of peptide binding to HSA calculated by the following equation:
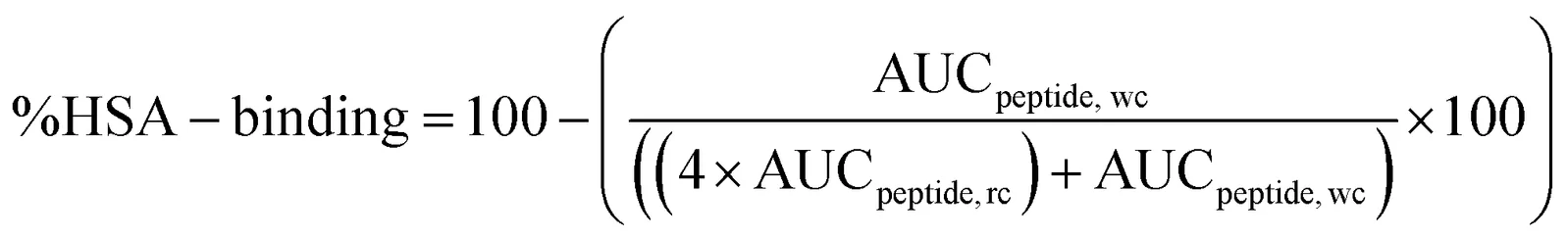
where AUC is area under the curve, rc is the red chamber, and wc is the white chamber.

### Statistical analysis

All data were analyzed using GraphPad Prism for Windows, versions 9 or 10 (GraphPad Software, San Diego, CA, USA).

## Author contributions

T. C.: investigation, data analysis, conceptualization, data analysis, visualization, methodology and writing the original draft. M. C.: investigation, visualization, methodology and writing the original draft. V. N.: supervision, methodology and resources. M. A. R. B. C.: supervision, methodology and resources. L. T.: methodology and resources. F. G.: methodology and resources. A. S.: investigation, methodology and writing the original draft. C. C.: conceptualization, supervision, methodology. K. R.: conceptualization, project administration, supervision, methodology, visualization, and writing the original draft.

## Conflicts of interest

There are no conflicts to declare.

## Supplementary Material

CB-007-D6CB00229C-s001

CB-007-D6CB00229C-s002

## Data Availability

The data supporting this article have been included as part of the supplementary information (SI). Any additional data is available on request from the corresponding author. Supplementary information including additional methods, results, HPLC and mass spectra is available. See DOI: https://doi.org/10.1039/d6cb00229c.
