# Supplementary material for: Multivalent peptides for blood–brain barrier translocation, cell penetration and microRNA-21 inhibition in glioblastoma
Source: RSC Chem Biol. 2026 Sep 1. Online ahead of print. doi: 10.1039/d6cb00229c (PMC13584092; doi:10.1039/d6cb00229c)
Supplement: Supplementary file 1 [file CB-OLF-D6CB00229C-s001.pdf]

Supplementary Information (SI) for RSC Chemical Biology.
This journal is © The Royal Society of Chemistry 2026

Supplementary Information.

Multivalent peptides for blood-brain barrier translocation, cell penetration and microRNA-21 inhibition in glioblastoma

Talhat Chaudhry [a,b], Marco Cavaco [c], Vera Neves [c], Miguel A R B Castanho [c], Lauren Tonge [a,b], Francesca Giuntini [a,b], Adam Schofield [d], Christopher R. Coxon [d], Kehinde Ross[a,b]

[a] School of Pharmacy and Biomolecular Sciences, Liverpool John Moores University, Liverpool, United Kingdom.

[b] Institute for Health Research, Liverpool John Moores University, Liverpool, United Kingdom.

[c] Fundação GIMM – Gulbenkian Institute for Molecular Medicine, Av. Prof Egas Moniz, 1649-028 Lisboa, Portugal.

[d] Faculdade de Medicina, Universidade de Lisbia, Av. Prof. Egas Moniz, 1649-028, Lisboa, Portugal.

[e] EaStChem School of Chemistry, The University of Edinburgh, Joseph Black Building, David Brewster Road, Edinburgh, EH9 3FJ, UK.

.

Correspondence
Kehinde Ross
School of Pharmacy and Biomolecular Sciences
Liverpool John Moores University
Liverpool
L3 3AF
Email: o.k.ross@ljmu.ac.uk

Supplementary Methods

Synthesis of Peptide Fusions

For tandem peptide fusions **20** and **21,** an Ahx-Lys-Ahx spacer sequence was incorporated between the CPP and the miRNA inhibiting peptide. The N-terminal amine was capped using acetic anhydride and the lysine residue in the Ahx-Lys-Ahx' spacer orthogonally protected using the acid/base stable allyloxycarbonyl (Alloc) protecting group. Peptides were purified to >95% purity using prep-HPLC, prepared as 2 mM stock solutions in dimethyl sulfoxide (DMSO) and frozen at -20℃ until use. The Alloc protecting group was selectively removed using palladium-tetrakis catalyst ($Pd(PPh_3)_4$, 0.1 eq.) and phenylsilane (20 eq.) in extra dry dichloromethane (DCM; 4 mL) under a N_2 inert atmosphere (3 x 30 min) shaking at room temperature. Finally, the resin was sequentially washed with 0.5% *N,N*-diisopropylethylamine (DIPEA) in *N,N*-dimethylformamide (DMF) followed by 0.5% sodium diethyldithiocarbamate and DCM only.

Cellular Fluorescence Quantification

Images were collected using a Zeiss LSM 710 confocal microscope using oil immersion objective 63x. For FITC, excitation wavelength of 488 nm (green laser), emission wavelength 493–602 nm and laser power 5% were used. For DAPI, excitation wavelength 405 nm (blue laser), emission wavelengths 410–496 nm and laser power 0.8% were employed. To quantify the cellular uptake of FITC labelled peptides, images for each sample were captured from 5 different fields of view to span at least 30 cells. Fluorescence intensity was quantified using image J. For each image, the channels were separated and the green channel was used for analysis. Cells were manually outlined using the freehand tool, the mean fluorescence, integrated density and area of each cell was measured. The same was repeated for the background, to subtract background fluorescence. To calculate the corrected total cellular fluorescence (CTCF) the equation below was used.

$$\text{CTCF} = \text{Integrated Density} - (\text{Area of selected cell} \times \text{Mean fluorescence of background readings})$$

Finally, the data obtained was normalised with respect to peptide **1** unless indicated otherwise.

Peptide Colocalisation studies

Cells were labelled with Lysotracker Red DND-99 to determine if peptides entered cells via the endocytotic pathway. Cells grown on glass coverslips in 6-well plates overnight, treated with the peptides (10 µM) and Lysotracker Red (LysRed DND-99). Subsequently, the cells were washed with PBS and treated with lysotracker red DND-99 (500 nM) in media for 1 h. Cells were washed with PBS and fixed using 4% paraformaldehyde (PFA) for 15 min at room temperature. After successive washes in PBS, the coverslips were then mounted onto glass slides using Fluoromount G containing DAPI. Images were acquired on a Zeiss LSM 710 confocal microscope.

Cell treatment with peptide uptake inhibitors

Cells were pre-treated with sodium azide (NaN_3) or methyl-β-cyclodextran (MβCD) to establish if peptide endocytosis was ATP dependent and to determine if peptide uptake was mediated by

caveolae mediated endocytosis. Cells were grown on glass cover slips overnight. The cells were then pre-treated with NaN_3 (10 mM) or MβCD (5 mM) and incubated for 1 h. Cells were washed with PBS, treated with the relevant peptides in complete media for 24 h then fixed in MeOH. Peptide fluorescence was quantified as outlined above.

Peptide Stability in Serum

To evaluate the stability of the peptides in human serum (HS), aqueous HS (25% *v/v*) was equilibrated at 37°C for 15 min and spiked with the peptide of interest to a final concentration of 300 µM and final volume of 200 µL (in triplicate). Samples were incubated at 37°C while continuously mixing in a sample mixer (HulaMixer, orbital mixing at 20 rpm) for known time intervals (0, 15 min, 30 min, 45 min, 1 h, 2h, 4h, 8h and 24h). Reactions were quenched by the addition of methanol (800 µL, 1:5 ratio), leaving a final notional peptide concentration of 60 µM. The reaction mixtures were cooled on ice for at least 30 min to precipitate serum proteins and centrifuged at 800 x g for 5 min to pellet the precipitated proteins. The supernatant was analysed by reversed-phase HPLC. A blank control experiment was carried out under the same conditions, but the peptide stock solution was replaced with DMSO. The concentration of intact peptide at time zero was taken as 100% and concentrations were measured against a calibration curve, plotted for the respective peptide. For the calibration curve, aqueous HS (25% *v/v*) was quenched using methanol (1:5 ratio) and centrifuged. The blank supernatant was taken and known concentrations of peptide were added (100, 75, 60, 50, 40, 25,10 and 5 µM). All experiments were performed in at least triplicate and on different days. All samples were analysed on Agilent 1200 Series HPLC comprising a diode-array detector (215/ 280 nm).

Circular Dichroism

Circular dichroism (CD) spectra of the lead peptides were acquired in a J-815 spectropolarimeter (Jasco, Japan) at 25 °C in the 190-260 nm wavelength range, with a bandwidth of 1 nm and a scan speed of 50 nm min^{-1}, using a 0.1 cm quartz cell. The final spectra for each peptide were the average of ten consecutive scans per sample after subtraction of buffer baselines. Results were expressed as mean residue ellipticity ($[\theta]_{MRW}$) (deg ´ cm^2 ´ $dmol^{-1}$), as follows:

$$[\theta]_{MRW} = \frac{\theta_{Obs} \times MRW}{10dc}$$

where, Θ_{obs} is the observed ellipticity in degrees, MRW is the mean residue weight, d is the cell path length and c is the peptide molar concentration.

Viability Assay – CellTiter-Blue®

Peptide cytotoxicity on HBECi cells used for the was determined using CellTiter-Blue® cell viability assay following a protocol previously optimized (1). Briefly, cells were allowed to grow until 80% confluence in a culture T-flask. Then, they were carefully harvested with trypsin-EDTA (Gibco/Thermo Fisher, USA) and seeded (10 000 cells/well) into pre-coated with attachment factor protein solution (Gibco/Thermo Fisher, USA) 96-well clear flat-bottomed polystyrene plates (Corning, USA) for 24 h. Subsequently, they were washed two times with 1x PBS and one time with DMEM:F12 medium, and 100 µL of previously diluted samples (range from 0.2 – 100.0 µM) in DMEM:F12 medium were added to cells. After 24 h, cells were washed two times with 1x PBS and one time with DMEM:F12 medium

and 20 µL of CellTiter-Blue® Reagent (diluted in 100 µL of DMEM:F12 medium) was added to each well and incubated for 2h in culturing conditions.

Peptides translocation was determined by fluorescence intensity (λ_{ex} = 495 nm, λ_{em} = 519 nm), using a Varioskan™ LUX multimode microplate reader. Samples recovery (%) were calculated as follows:

$$Recovery\ (\%) = \frac{F_i - F_{cells}}{F_{sample} - F_{Medium}} \times 100$$

where, F_i is the fluorescence intensity of samples recovered, F_{cells} is the intrinsic fluorescence intensity of cells without samples incubation, F_{sample} is the intensity of total sample initially added to the transwell apical side, and F_{Medium} is the intensity of DMEM:12 medium without phenol red. Experiments were performed in triplicates on different days using three independently grown cell cultures.

In vitro BBB Integrity Assay

After the *in vitro* BBB translocation assay, cells were washed two times with 1x PBS and one time with DMEM:F12 medium without phenol red, and integrity of the barrier evaluated using tetramethylrhodamine isothiocyanate-4 kDa dextran (RD4) (Sigma-Aldrich, Spain), previously diluted in DMEM:F12 medium without phenol red to an absorbance below 0.1, for 2 h.

$$RD4\ Permeability(\%) = \frac{F_i - F_{cells}}{F_{RD4} - F_{Medium}} \times 100$$

where, F_i is the fluorescence intensity of samples recovered, F_{cells} is the intrinsic fluorescence intensity of cells without samples incubation, F_{RD4} is the intensity of total RD4 initially added to the transwell apical side, and F_{Medium} is the intensity of DMEM:12 medium without phenol red. The integrity of the barrier is indirectly proportional to the percentage of RD4 recovered and was determined using the following equation:

$$Recovery\ (\%) = 100 - RD4\ Permeability\ (\%)$$

Experiments were performed in triplicates on different days using three independently grown cell cultures.

Viability Assay - Alamar Blue

Cells were seeded in triplicate 10,000 cells per well of a 96-well plate, in 200 mL of media and incubated for 24 h (37 °C, 5% CO2). peptides of interest (5 to 100 mM) were added, positive control cells were treated with 40% DMSO and negative control cells were left untreated (100% viability). A mock treatment was also included, i.e., media was supplemented with DMSO so that the final DMSO concentration in the well was equivalent to that in the peptide treated wells. After 24 h, media were aspirated and cells washed with PBS and treated with resazurin (0.1 mg mL^{-1}; 100 µL) in the dark (wrapped in foil) at 37°C for 4 hours. Fluorescence was measured on a CLARIOstar plate reader (BMG labtech, UK), with an excitation wavelength of 545 nm and an emission wavelength of 600nm.

RNA Isolation

Cells were seeded at 100,000 cells per well of a 12-well plate. Peptides conjugates were applied to cells at a final concentration of 10 µM in complete medium for 24 h. Total RNA was extracted from

cell lysates using ReliaPrep™ miRNA Cell and Tissue Miniprep System (Promega, Southampton, UK), in accordance with the manufacturer's instructions. All centrifugation steps were performed at 12,000 x g at ambient temperature. Briefly, cells were washed using ice-cold PBS and lysed in the 12-well or 24-well plates using 175 or 150 µL of lysis buffer (supplemented with 2% 1-Thioglycerol). The lysate was mixed with RNA Dilution Buffer (RDB) and centrifuged. The cleared homogenate was transferred to a microcentrifuge tube, mixed with 400 µL isopropanol and transferred to a ReliaPrep™ Minicolumn. The column was washed with RNA wash solution (RWA; 500 µL) twice and nucleic acids eluted with 40 µL nuclease-free water. Subsequently DNase I (5 µL) and DNase 10X Buffer (5 µL) were added to the eluate and the mixture incubated for 5 min at room temperature. Lysis buffer (150 µL) and 95% ethanol (300 µL) was added and the mixture vortexed, transferred to a clean ReliaPrep™ Minicolumn, and centrifuged. After washing the column twice with RWA, RNA was eluted in 20 µL nuclease-free water. Total RNA concentration was determined on a NanoDrop™ 2000c spectrophotometer (Thermo Fisher, UK), with optical densities (OD) of 260 and 280 nm, averaged from triplicate readings. The absorbance ratio between 260/280 nm was used to determine the quality of isolated RNA, if the ratio lied within a 1.9- 2.1 range it was indicative of good RNA quality. Isolated RNA was stored at -80 °C until required.

Reverse transcriptase PCR for analysis of miR-21 expression

Expression of miR-21-5p was assessed by real-time quantitative PCR (RT-qPCR). Total RNA was reverse transcribed into complementary DNA (cDNA) using miScript II reagents (Qiagen,Manchester, UK). A mastermix compromising 5x miScript HiFlex Buffer (4 µL), 10x miScript nucleic acid mix (2 µL) and miScript reverse transcriptase mix (2 µL) was prepared and scaled up depending on the sample size. The samples were prepared in RNase/DNase-free PCR tubes comprising mastermix (8 µL), 300 ng of template RNA and RNase-free water to a final volume of 20 µL. Samples were incubated at 37ºC for 60 min and heated to 95ºC for 5 min to inactive the miScript reverse transcriptase mix. The cDNA was diluted 10-fold in RNase-free water prior to qPCR experiments.

SUPPLEMENTARY FIGURE 1

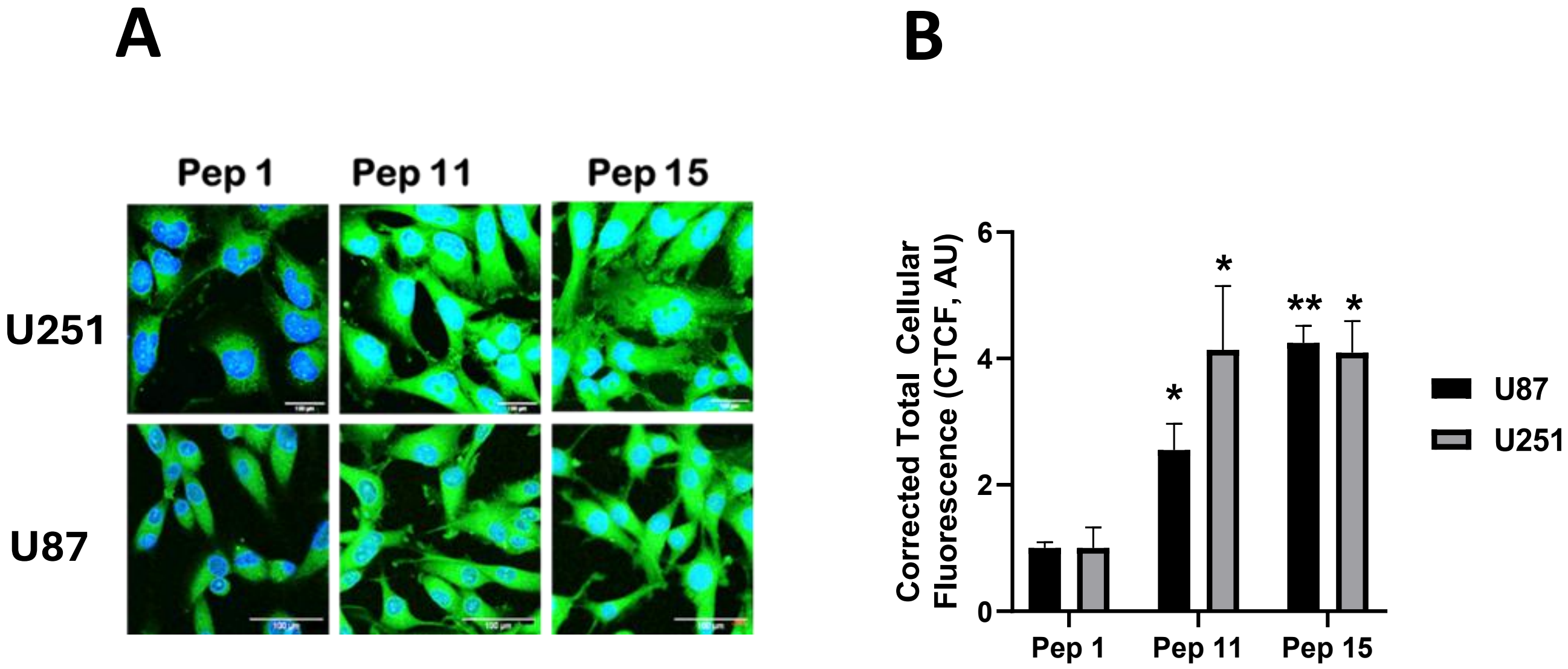

Supplementary Figure 1: Comparison of peptide uptake in U251 and U87 GBM cells. Cells were treated with 10 µM of peptide for 24 h at 37°C. (A) Internalisation of both **11** and **15** was raised compared to **1** in both U87 and U251 cells. **1 =** SYPGWSW, **11 =** S(Cha)PGWSW, **15** = S(Nal)PGWSW. The U87 images are the same as shown in Fig 1A and are included here for comparison. (B) Quantitative analysis of confocal micrographs spanning in 30 cells confirmed statistically significant increase in peptide **11** and **15** uptake compared to **1** . Error bars represent SEM; statistical analysis carried out using one-way ANOVA with Dunnett's multiple comparisons test: levels of significance *P < 0.1, **P < 0.01.

.

SUPPLEMENTARY FIGURE 2

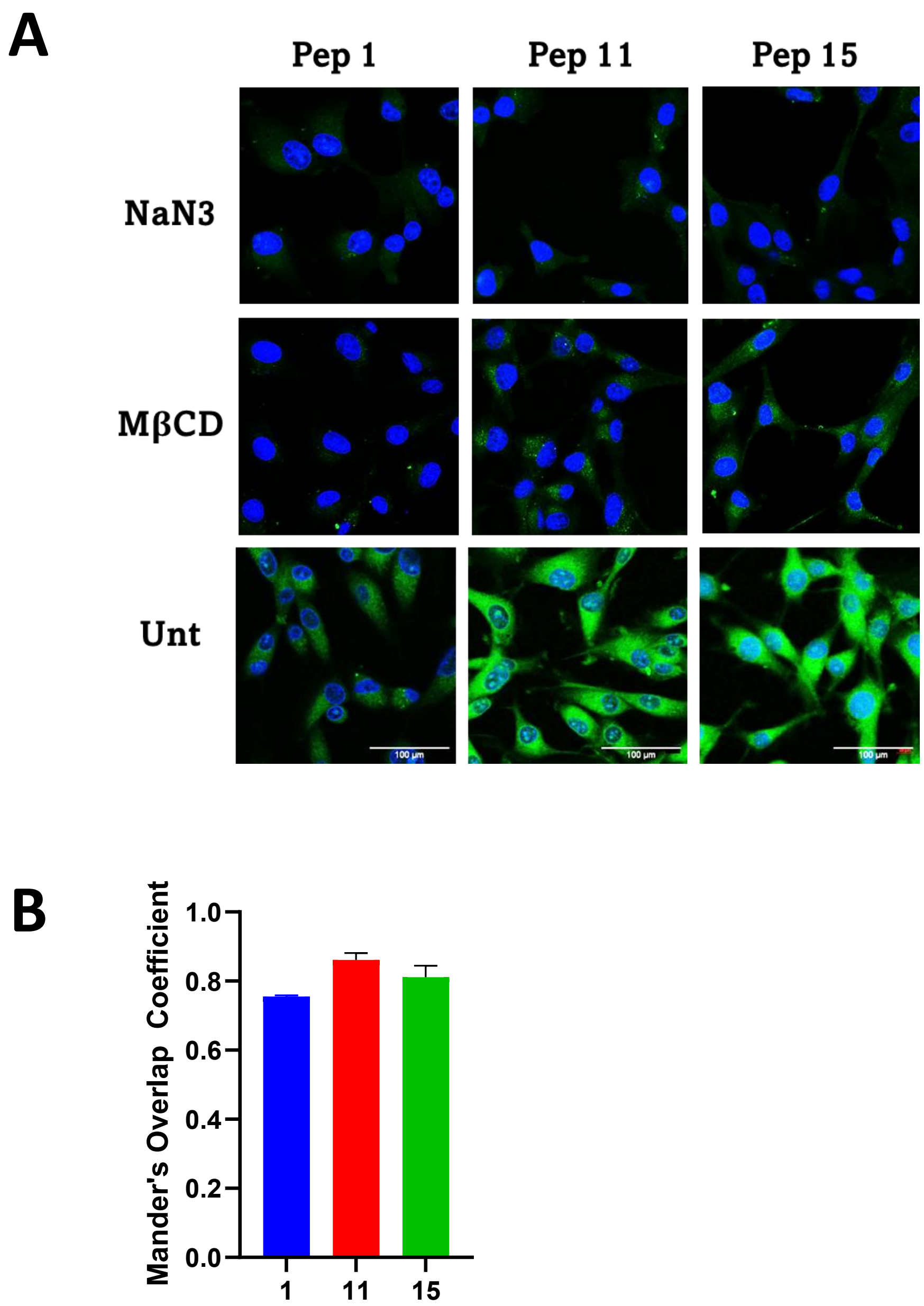

Supplementary Figure 2: Mechanism of peptide internalisation in U87 cells. (A) Confocal micrographs showing minimal uptake of peptides **1**, **11** and **15.** Cells were pre-incubated for 1 hour with NaN_3 (10 mM) or MβCD (5 mM) or left untreated (Unt). Subsequently, FITC-labelled peptides prepared in complete medium were applied to cells at 10 µM for 24 h. Cells were fixed in methanol and nuclei stained with DAPI. Confocal micrographs were acquired with a 63x objective. *Scale bar* = 100 µm. (B) Endosomes were labelled with Lysotracker Red DND-99 and co-localisation of peptides **1, 11** and **15,** respectively, estimated from confocal micrographs. There was no significant different in the Manders overlap coefficient between the samples (one-way ANOVA with Tukey's post-test). Error bars represent SEM; n=2.

SUPPLEMENTARY FIGURE 3

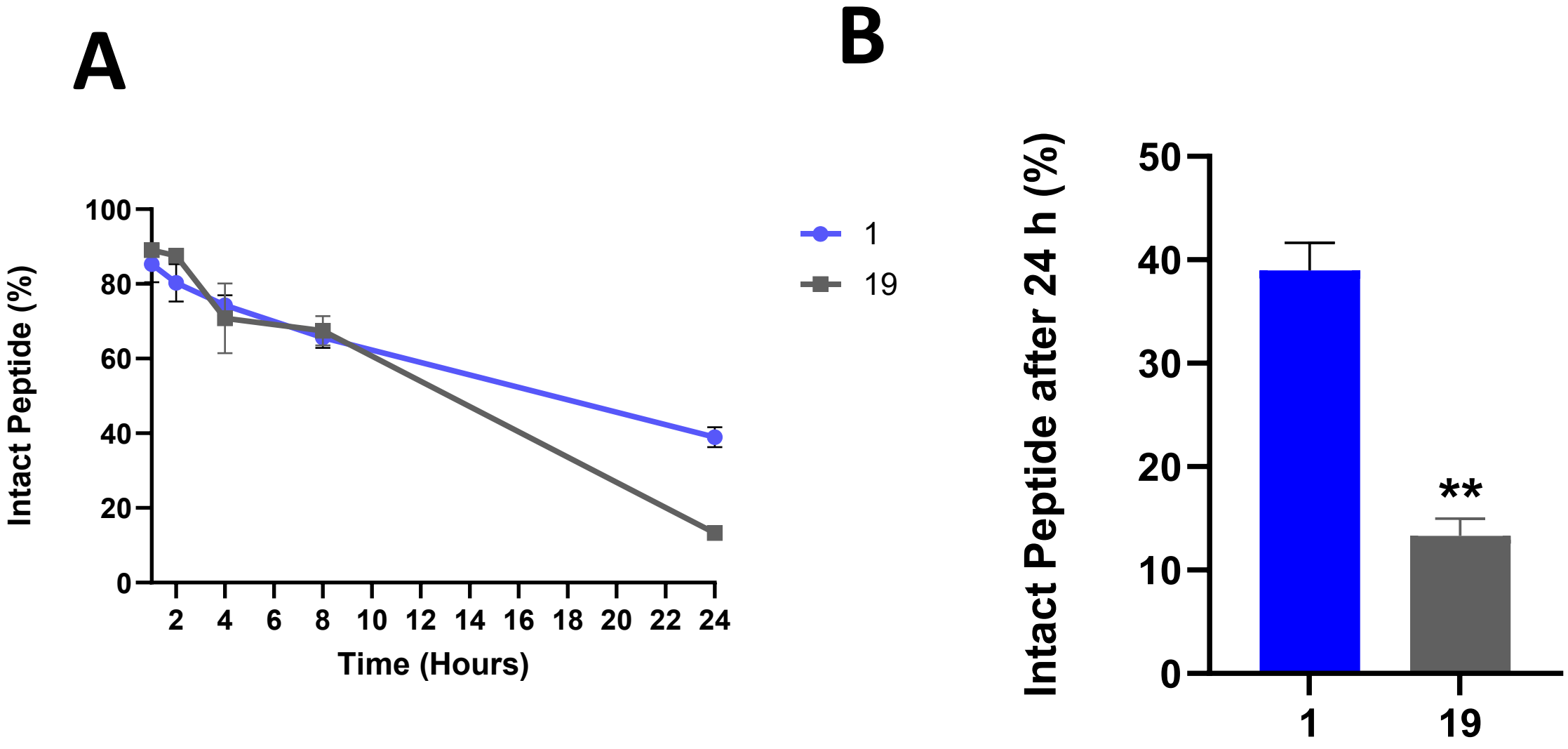

Supplementary Figure 3: Effects of the *N*-terminal FITC of peptide stability. (A) Degradation profile for peptide **1** compared to peptide **19** in diluted serum (25% v/v) over a 24 h period. Peptide degradation profile following 1 to 24 h of exposure to serum. Peptides **1** FTIC-Ahx-SYPGWSW and **19** (Ahx)SYPGWSW. The data was normalised to the concentration of peptide at time 0 (taken as 100%). The peptides were exposed to 25% human serum diluted in PBS. Peptide stability was determined at 9 different time intervals from 0 to 24 h by HPLC. Experiments were performed in triplicate. Error bars represent SEM. Error bars not shown are smaller than symbols. (B) Comparison of the degradation of the **1** and **19** after 24 h of exposure to diluted serum (25% v/v). All experiments were performed in triplicates. Error bars represent SEM; statistical analysis carried out with Student's t test. *** , P* < 0.005.

SUPPLEMENTARY FIGURE 4

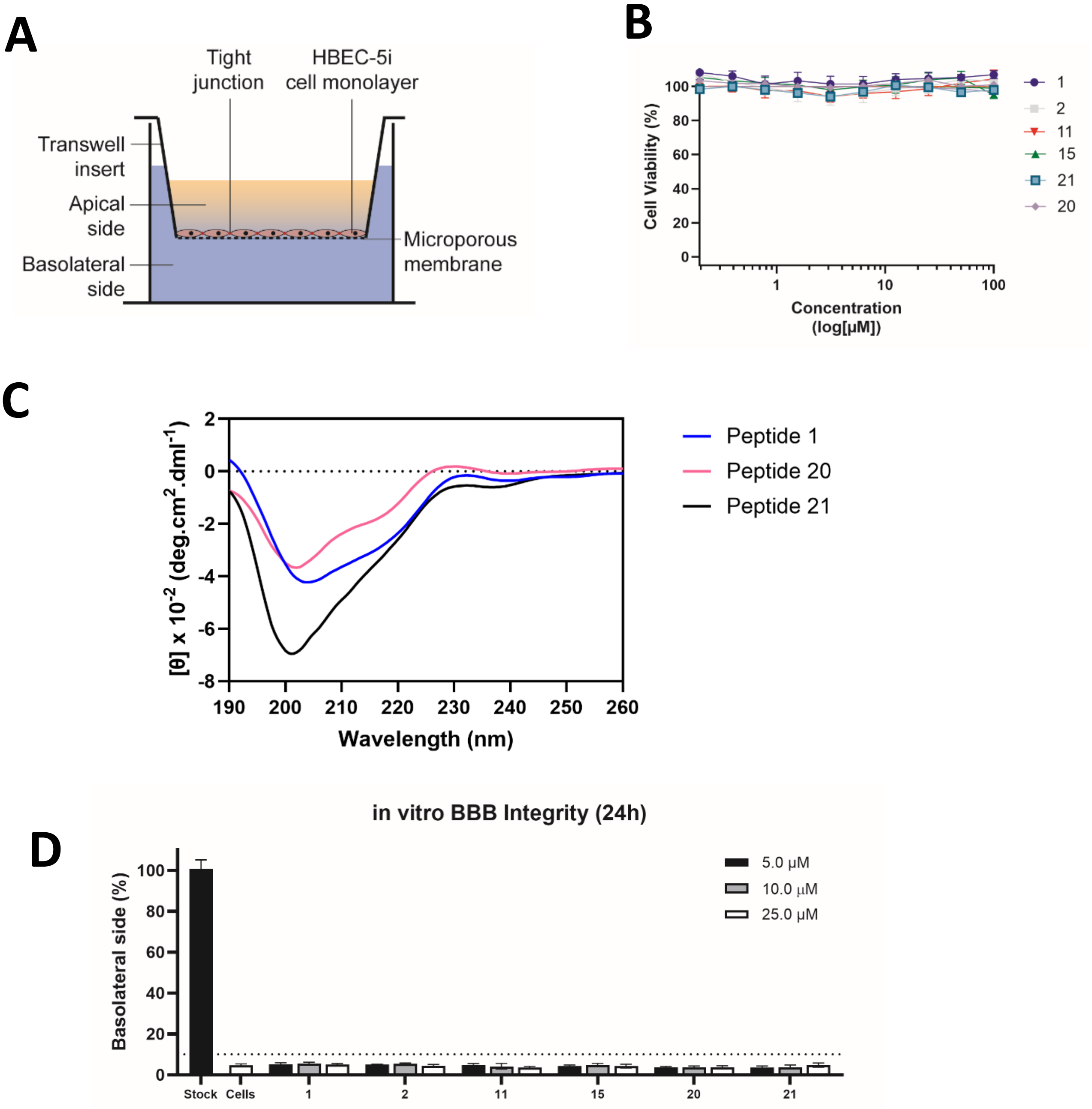

Supplementary Figure 4: Peptide translocation across the BBB**.** (A) Depiction of in vitro BBB model. **(B)** Viability of HBEC-5i cells following exposure to the indicated peptides (conc. 0.2- 100 µM). Peptides: (**1**) SYPGWSW, (**2**) SWYGPSW, (**11**) S(Cha)PGWSW, (**15**) S(Nal)PGWSW, (**20**) SYPGWSW-Ahx-K(FITC)-Ahx-ALWPPNLHAWVP and (**21**) SYPGWSW-Ahx-K(FITC)-Ahx-LPPAPWNWAHVL. Cell viability assays was performed using CellTiter-Blue®. Cells were incubated with the peptides for 24 h. Experiments were formed in triplicate on different days using three independently grown cell cultures. Error bars represent SEM. (C) Comparison of the secondary structure of **1**, **20** and **21** as revealed by circular dichroism. (D) Determination of transwell *in-vitro* BBB integrity. Barrier integrity was determined using tetramethylrhodamine isothiocyanate-4 kDa dextran (RD4) after the peptide translocation studies. The RD4 stock solution treatment serves as a positive control and negative control is HBEC-5i cells without exposure to peptide. Experiments were performed in triplicates. Error bars represent SEM. Similar data were obtained at the 1 h and 8 h timepoints (not shown).
