# Supplementary material for: Multivalent peptides for blood–brain barrier translocation, cell penetration and microRNA-21 inhibition in glioblastoma
Source: RSC Chem Biol. 2026 Sep 1. Online ahead of print. doi: 10.1039/d6cb00229c (PMC13584092; doi:10.1039/d6cb00229c)
Supplement: Supplementary file 2 [file CB-OLF-D6CB00229C-s002.pdf]

Supplementary Information (SI) for RSC Chemical Biology.
This journal is © The Royal Society of Chemistry 2026

Multivalent peptides for blood-brain barrier translocation, cell penetration and microRNA-21 inhibition in glioblastoma

Talhat Chaudhry [a,b], Marco Cavaco [c], Vera Neves [c], Miguel A R B Castanho [c], Lauren Tonge [a,b], Francesca Giuntini [a,b], Adam Schofield [d], Christopher R. Coxon [d], Kehinde Ross[a,b]

[a]School of Pharmacy and Biomolecular Sciences, Liverpool John Moores University, Liverpool, United Kingdom.

[b]Institute for Health Research, Liverpool John Moores University, Liverpool, United Kingdom

[c]Instituto de Medicina Molecular João Lobo Antunes, Faculdade de Medicina, Universidade de Lisboa, Av. Prof Egas Moniz, 1649-028 Lisboa, Portugal.

[d] EaStChem School of Chemistry, The University of Edinburgh, Joseph Black Building, David Brewster Road, Edinburgh, EH9 3FJ, UK

.

Correspondence

Kehinde Ross

School of Pharmacy and Biomolecular Sciences

Liverpool John Moores University

Liverpool

L3 3AF

Email: o.k.ross@ljmu.ac.uk

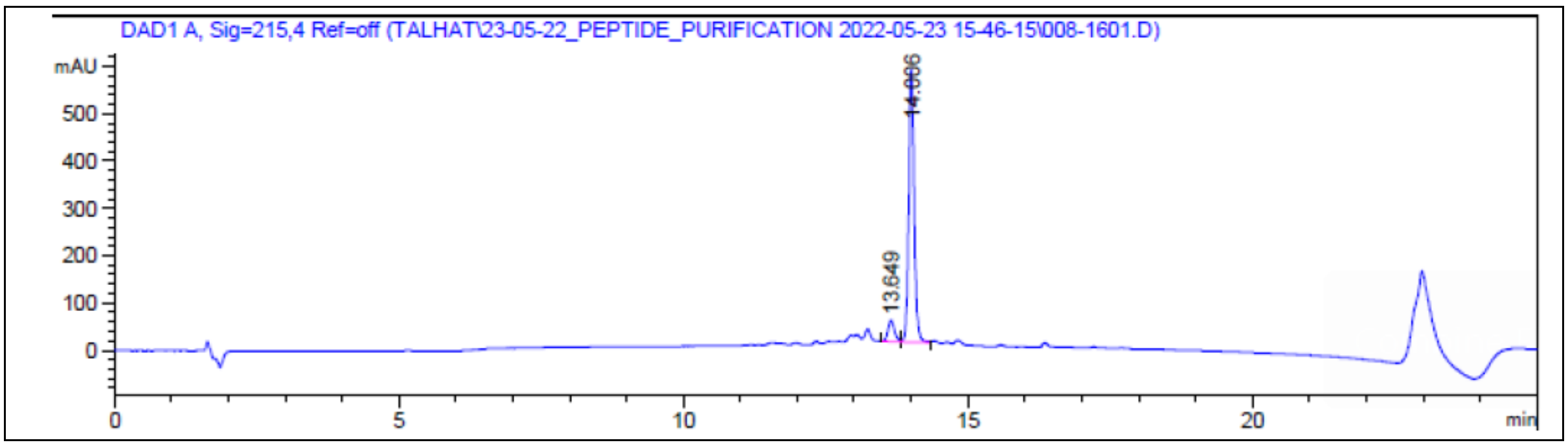

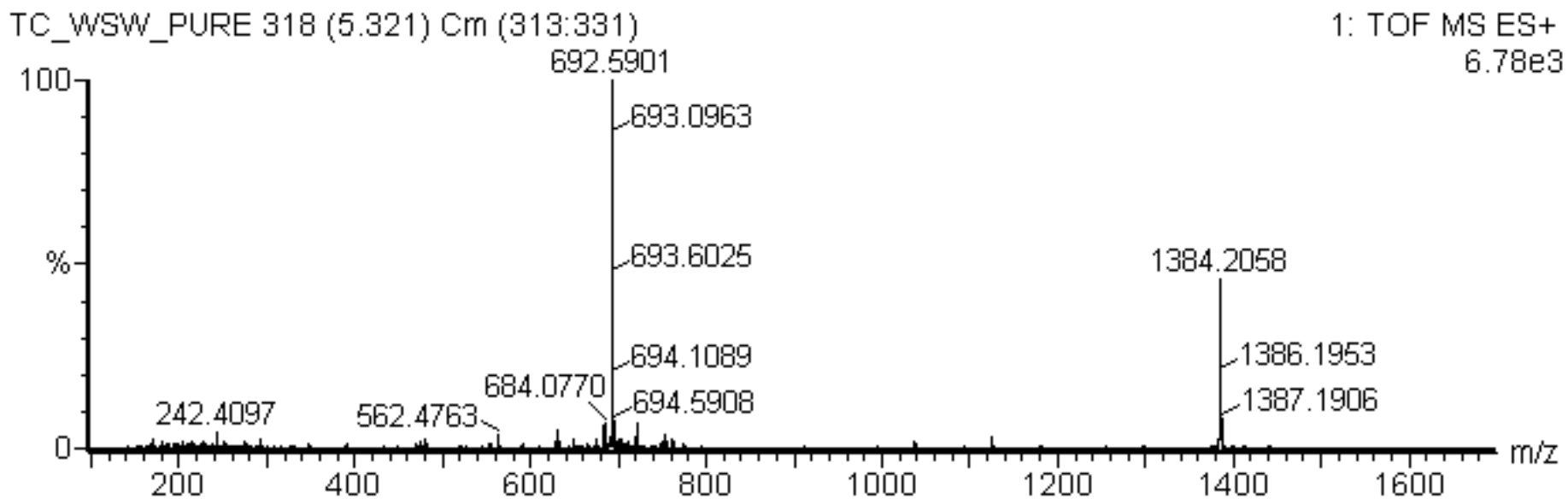

Figure S1: Peptide 1 (FITC-Ahx-SYPGWSW) purification data. Peptide purity > 95%. *Top*, HPLC spectra at 215 nm retention time 10.958 minutes. *Bottom*, HPLC spectra at 280 nm retention time 10.958 minutes. (B) LC-MS data for WSW-Wild Type (FITC-Ahx-SYPGWSW). Extracted mass spectra showed expected mass-to-charge ratios: m/z 1383 [M+H]+ and 692 [M+2H]+

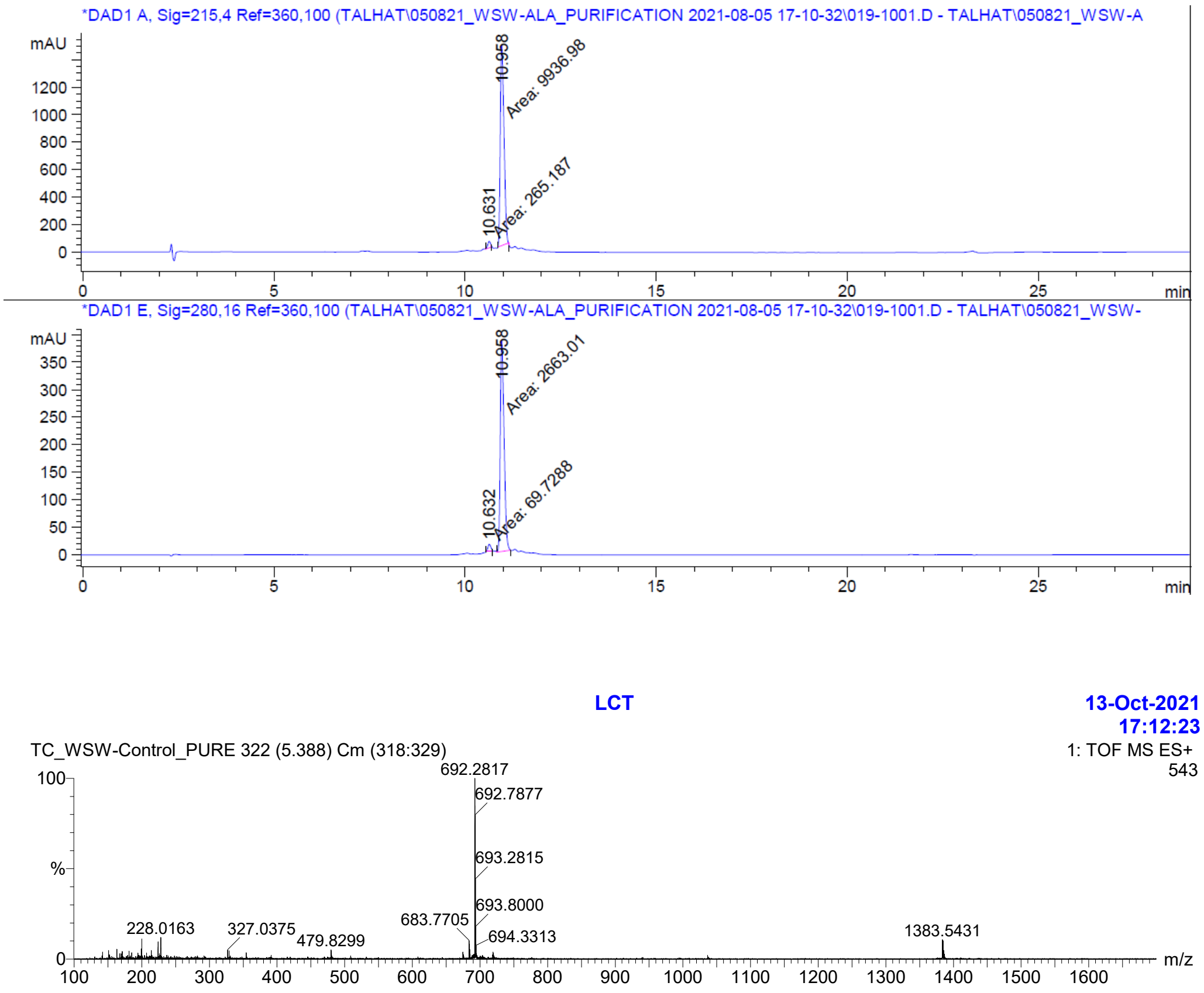

Figure S2. Peptide **2** (FITC-Ahx-SWYGPSW) purification data. (A) Peptide purity > 95%. *Top*, HPLC spectra at 215 nm retention time 10.958 minutes. *Bottom*, HPLC spectra at 280 nm retention time 10.958 minutes. (B). LC-MS data. Extracted mass spectra showed expected mass-to-charge ratios: m/z 1383 $[M+H]^+$ and 692 $[M+2H]^+$.

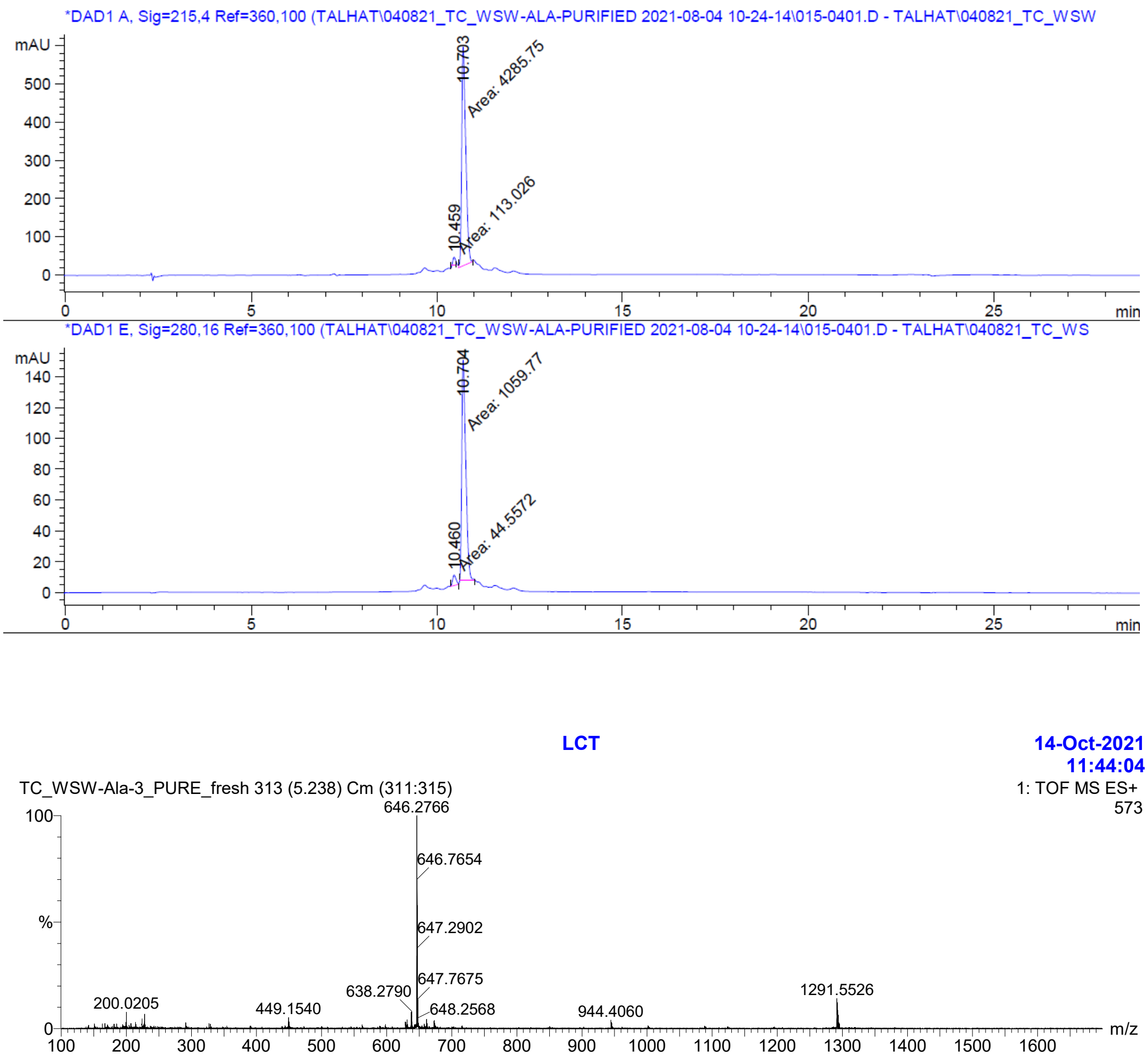

Figure S3. Peptide 3 (FITC-Ahx-SAPGWSW) purification data. (A) Peptide purity > 95%. *Top*, HPLC spectra at 215 nm retention time 10.703 minutes. *Bottom*, HPLC spectra at 280 nm retention time 10.704 minutes (B). LC-MS data. Extracted mass spectra showed expected mass-to-charge ratios: m/z 1291 $[M+H]^+$ and 646 $[M+2H]^+$.

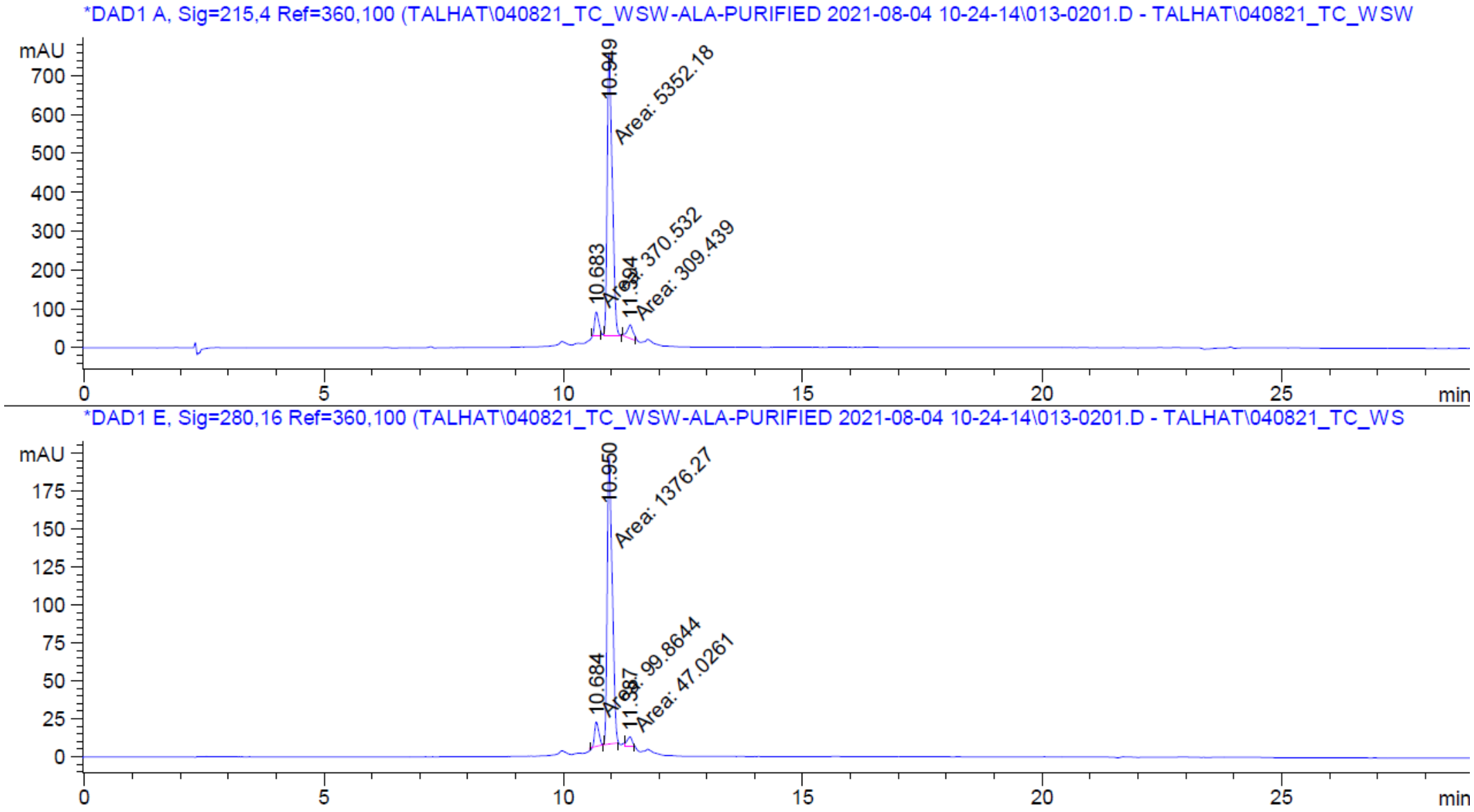

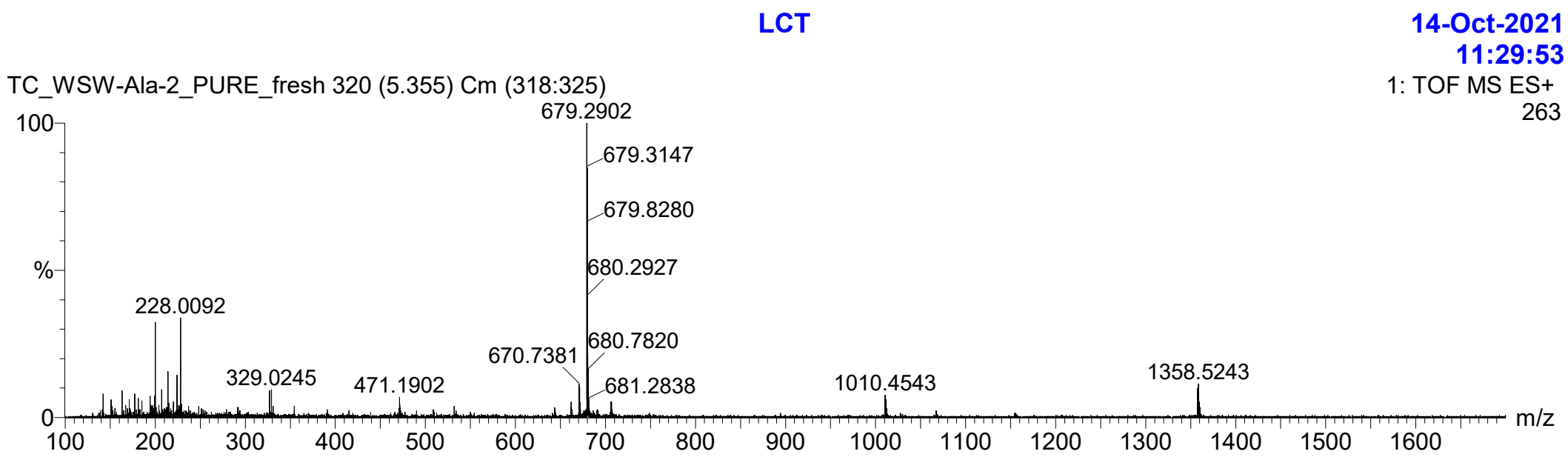

Figure S4. Peptide **4** (FITC-Ahx-SYAGWSW) purification data. (A) Peptide purity > 95%. *Top*, HPLC spectra at 215 nm retention time 10.949 minutes. *Bottom*, HPLC spectra at 280 nm retention time 10.950 minutes. (B) LC-MS data. Extracted mass spectra showed expected mass-to-charge ratios: m/z 1358 $[M+H]^+$ and 679 $[M+2H]^+$.

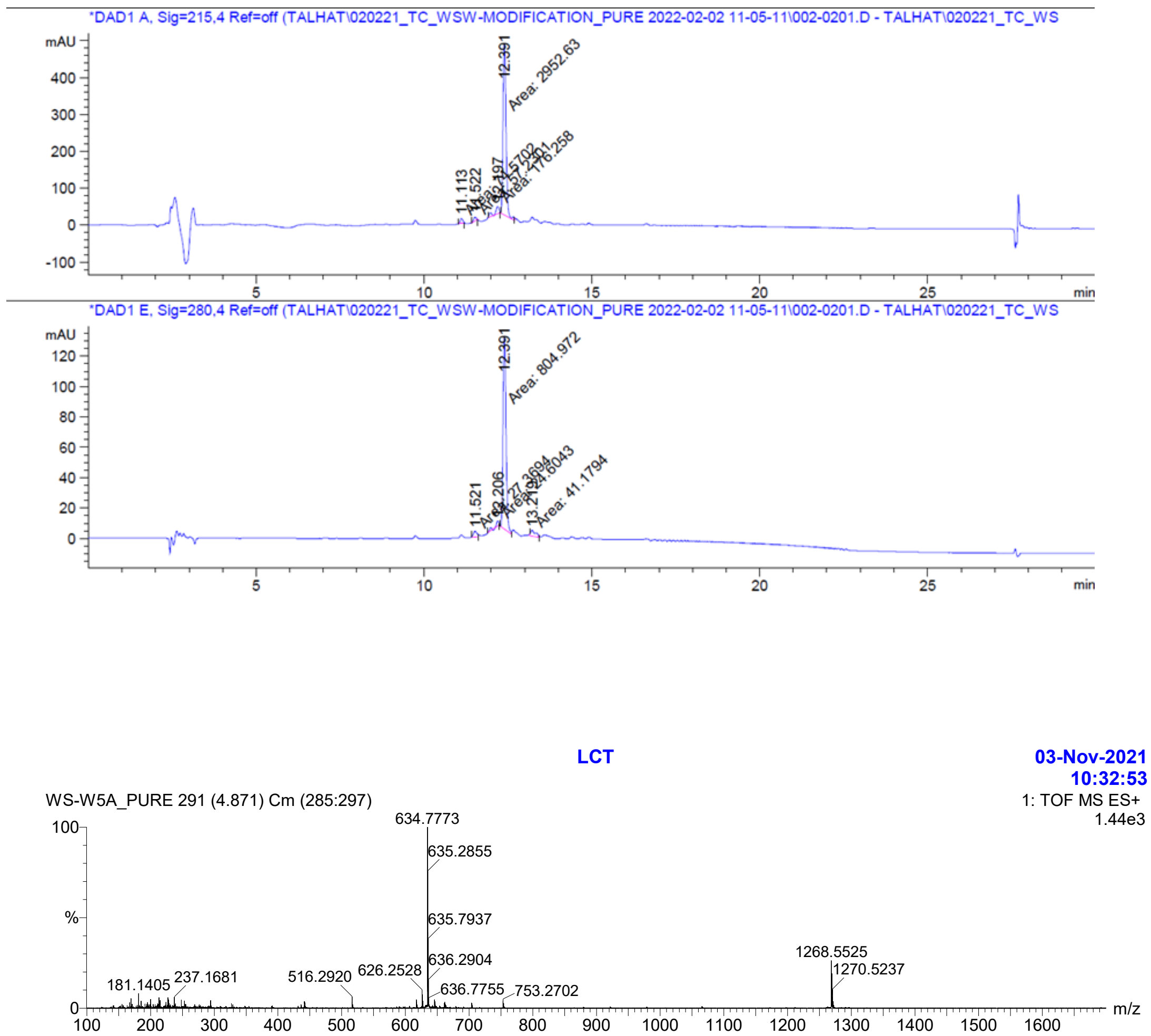

Figure S5. Peptide 5 (FITC-Ahx-SYPGASW) purification data. (A) Peptide purity > 95%. *Top*, HPLC spectra at 215 nm retention time 12.391 minutes. *Bottom*, HPLC spectra at 280 nm retention time 12.391 minutes. (B) LC-MS data. Extracted mass spectra showed expected mass-to-charge ratios: m/z 1268 $[M+H]^+$ and 634 $[M+2H]^+$.

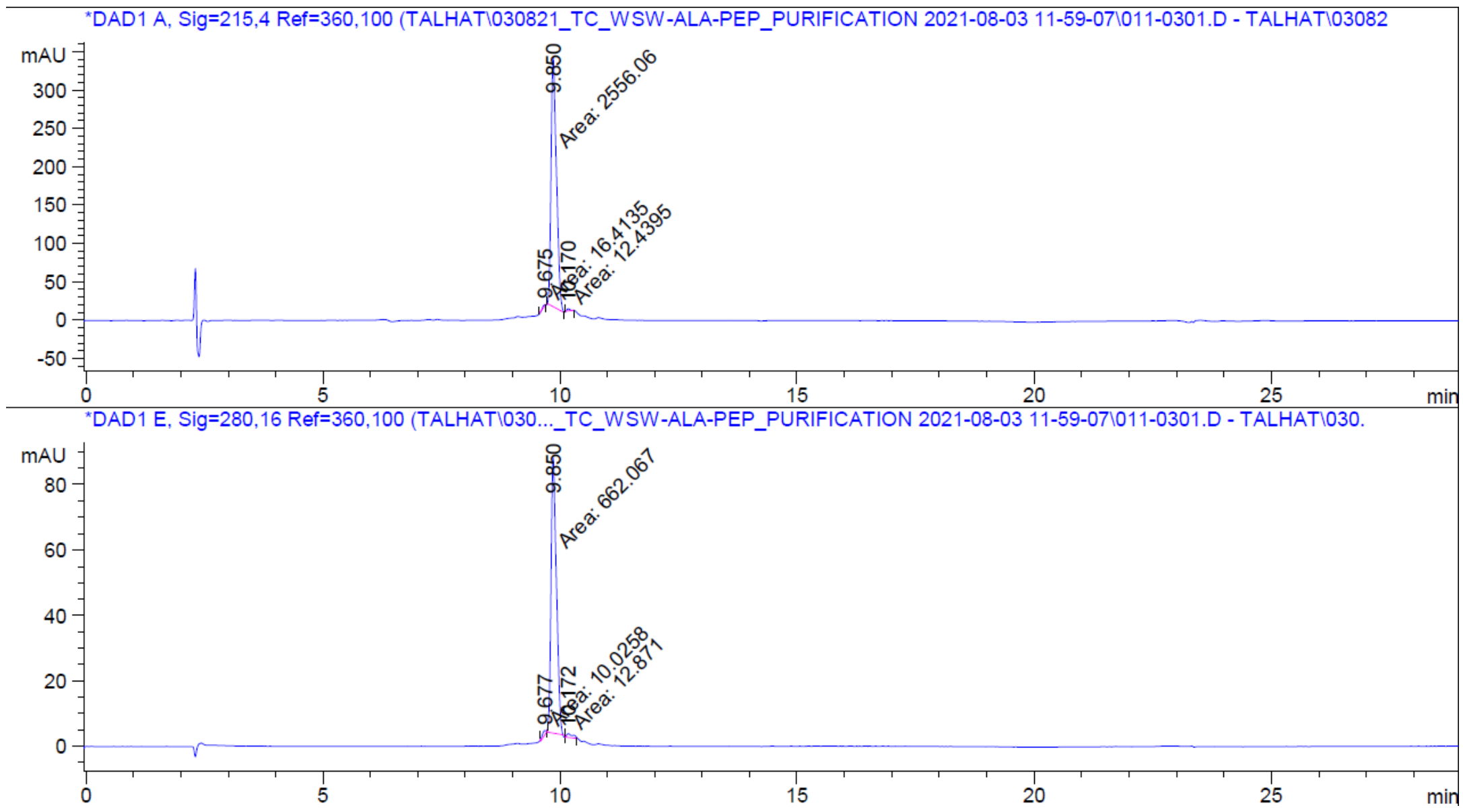

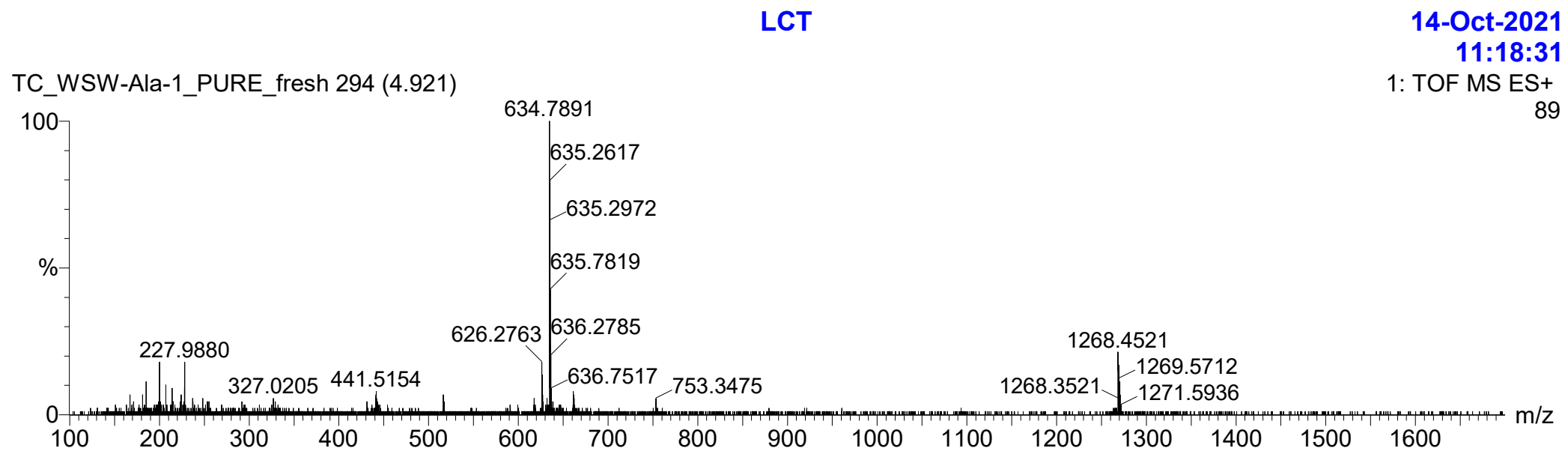

Figure S6. Peptide **6** (FITC-Ahx-SYPGWSA) purification data.(A) Peptide purity > 95%. *Top*, HPLC spectra at 215 nm retention time 9.850 minutes. *Bottom*, HPLC spectra at 280 nm retention time 9.850 minutes. (B). LC-MS data. Extracted mass spectra showed expected mass-to-charge ratios: m/z 1268 $[M+H]^+$ and 634 $[M+2H]^+$.

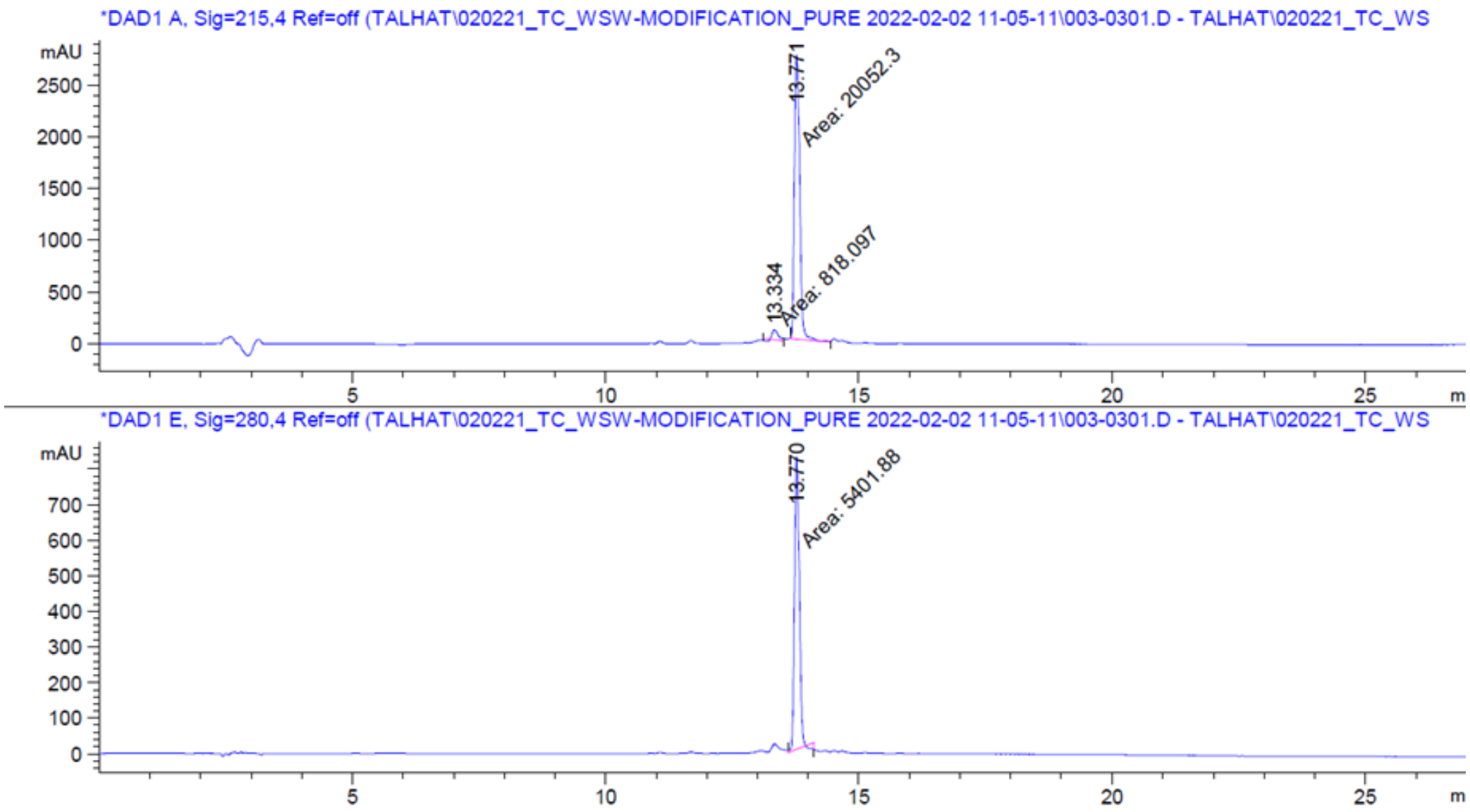

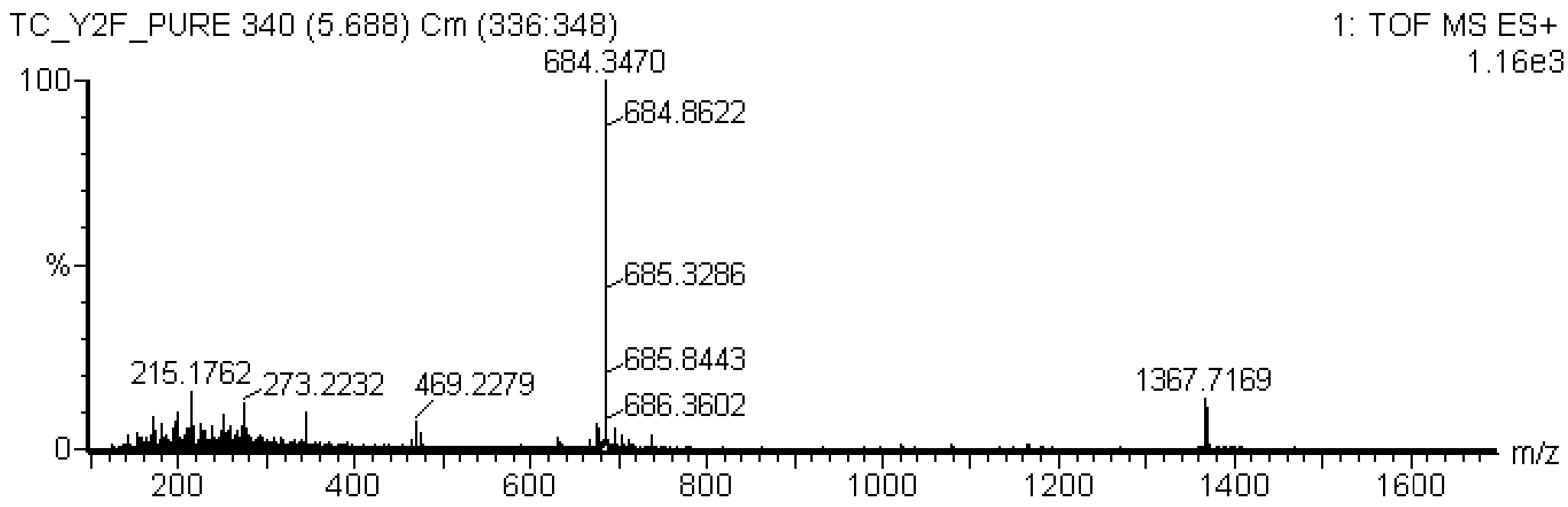

Figure S7: Peptide **7** (FITC-Ahx-SFPGWSW) purification data. (A) Peptide purity > 95%. *Top*, HPLC spectra at 215 nm retention time 13.771 minutes. *Bottom*, HPLC spectra at 280 nm retention time 13.770 minutes. (B) LC-MS data. Extracted mass spectra showed expected mass-to-charge ratios: m/z 1367 $[M+H]^+$ and 684 $[M+2H]^+$.

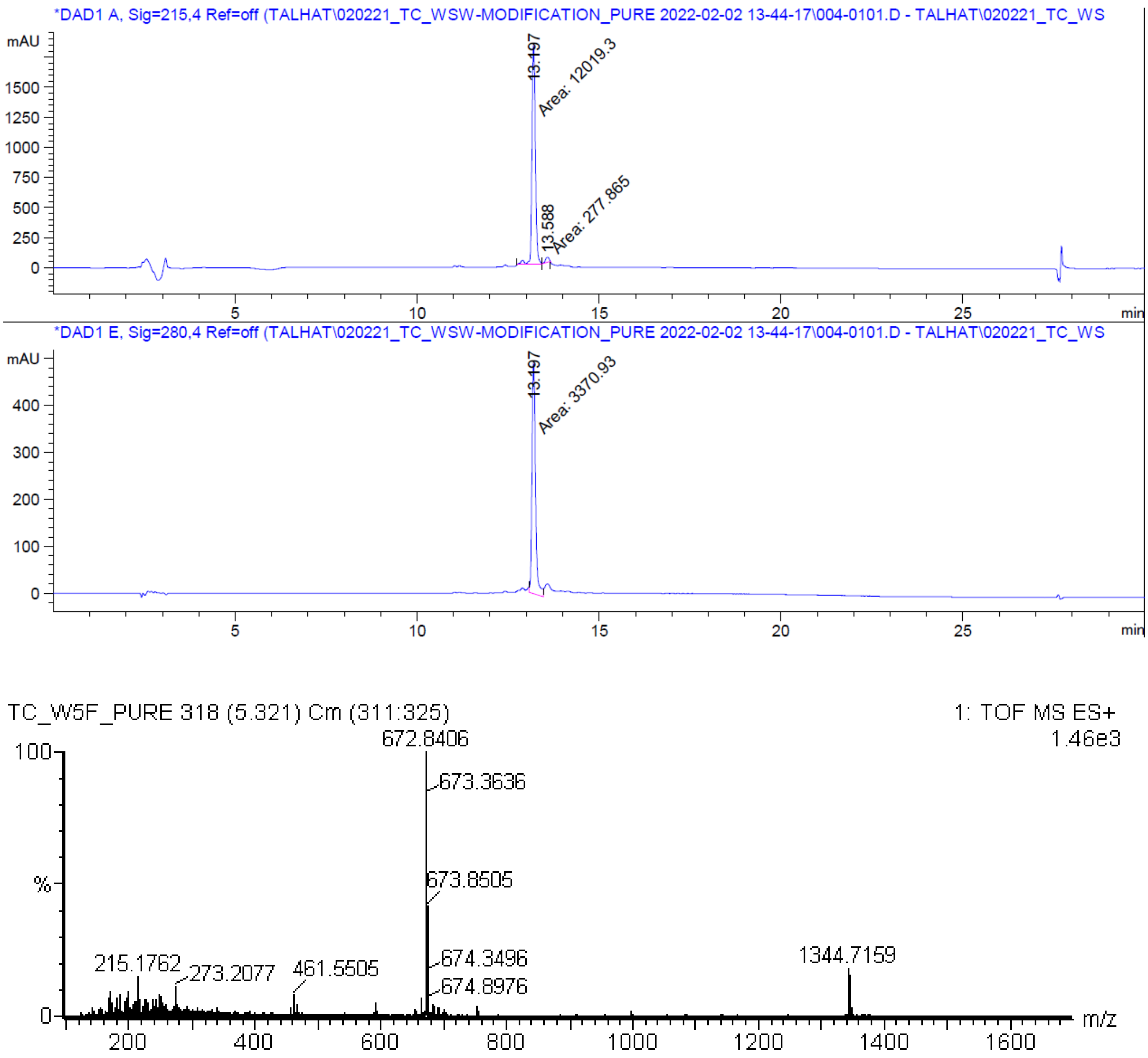

Figure S8: Peptide **8** (FITC-Ahx- SYPGFSW) purification data. (A) Peptide purity > 95%. *Top*, HPLC spectra at 215 nm retention time 13.197 minutes. *Bottom,* HPLC spectra at 280 nm retention time 13.197 minutes. (B) LC-MS data. Extracted mass spectra showed expected mass-to-charge ratios: m/z 1344 $[M+H]^+$ and 672 $[M+2H]^+$.

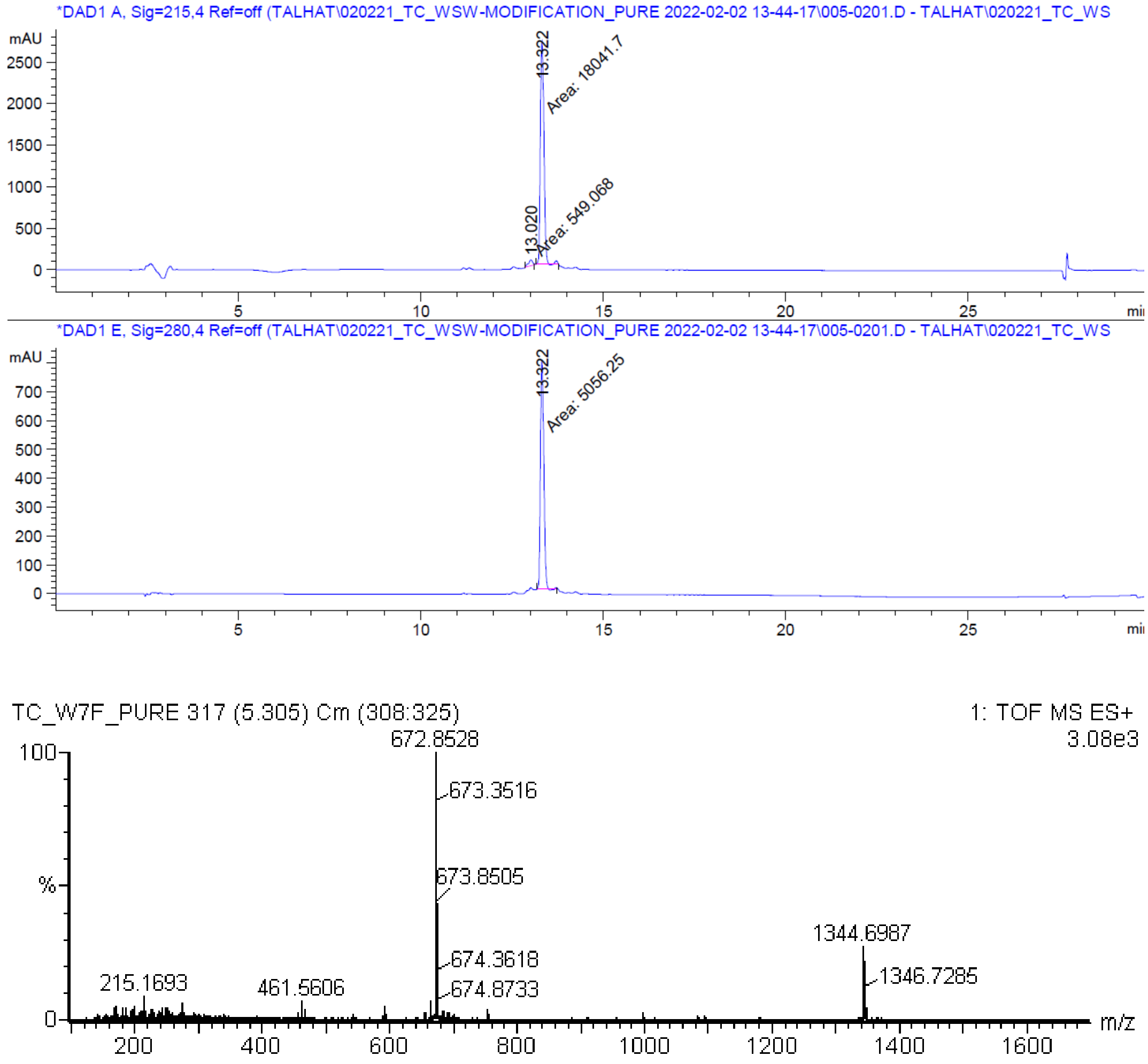

Figure S9. Peptide **9** (FITC-Ahx- SYPGWSF) purification data. (A) peptide purity > 95%. *Top*, HPLC spectra at 215 nm retention time 13.322 minutes. *Bottom*, HPLC spectra at 280 nm retention time 13.322 minutes. (B) LC-MS data. Extracted mass spectra showed expected mass-to-charge ratios: m/z 1344 [M+H]$^+$ and 672 [M+2H]$^+$.

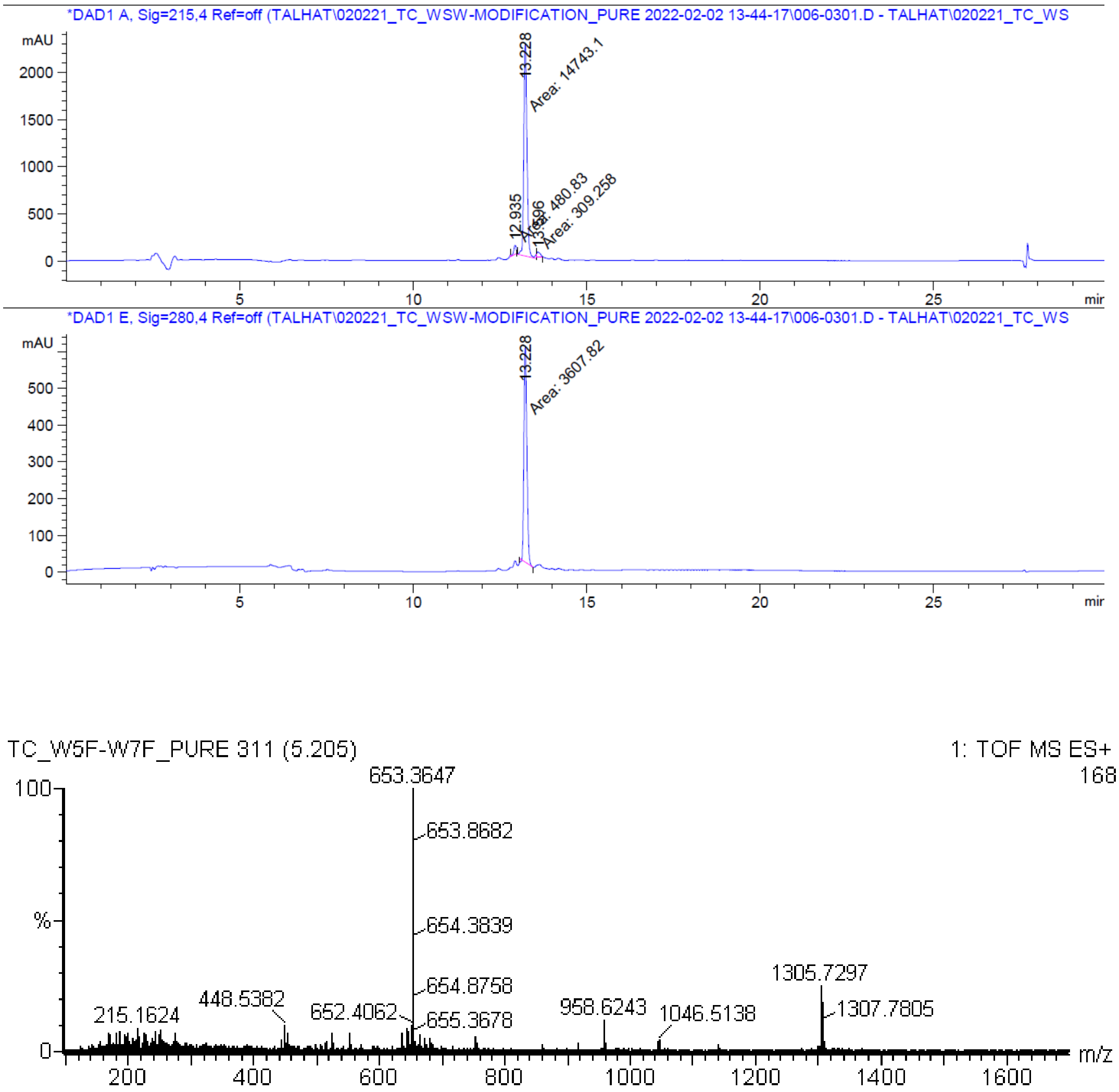

Figure S10. Peptide **10** (FITC-Ahx- SYPGFSF) purification data. (A) Peptide purity > 95%. *Top*, HPLC spectra at 215 nm retention time 13.228 minutes. *Bottom*, HPLC spectra at 280 nm retention time 13.228 minutes. (B) LC-MS data. Extracted mass spectra showed expected mass-to-charge ratios: m/z 1305 [M+H]+ and 653 [M+2H]+.

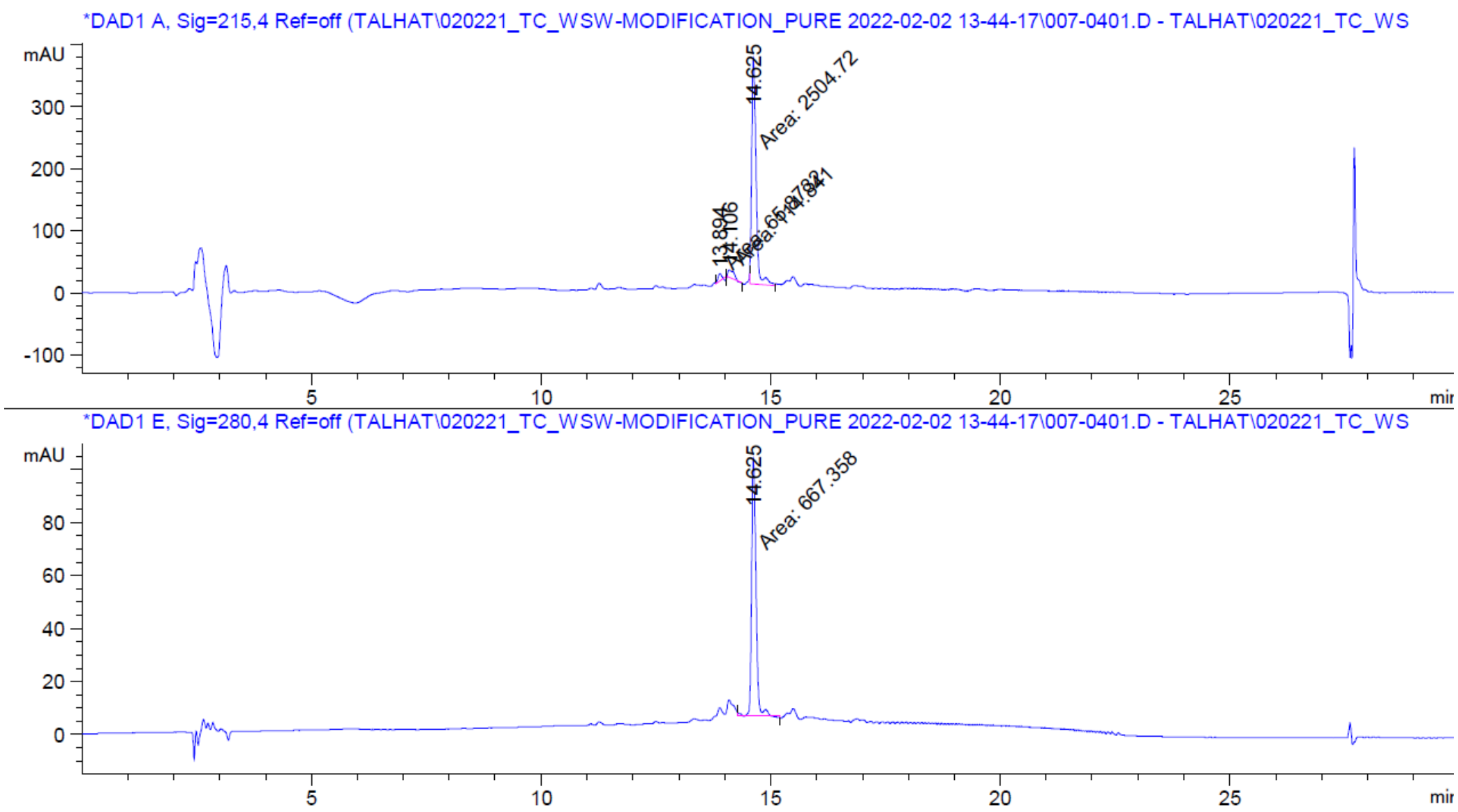

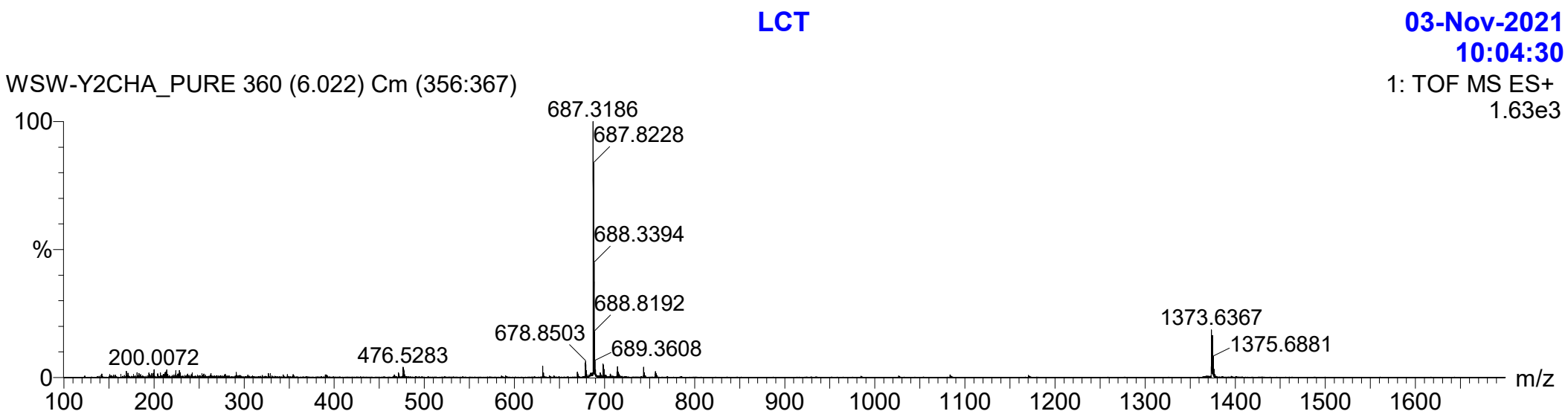

Figure S11. Peptide **11** (FITC-Ahx- S(Cha)PGWSW) purification data (A), peptide purity 93%. *Top,* HPLC spectra at 215 nm retention time 14.625 minutes. *Bottom*, HPLC spectra at 280 nm retention time 14.625 minutes. (B) Peptide 29. LC-MS data. Extracted mass spectra showed expected mass-to-charge ratios: m/z 1373 $[M+H]^+$ and 687 $[M+2H]^+$.

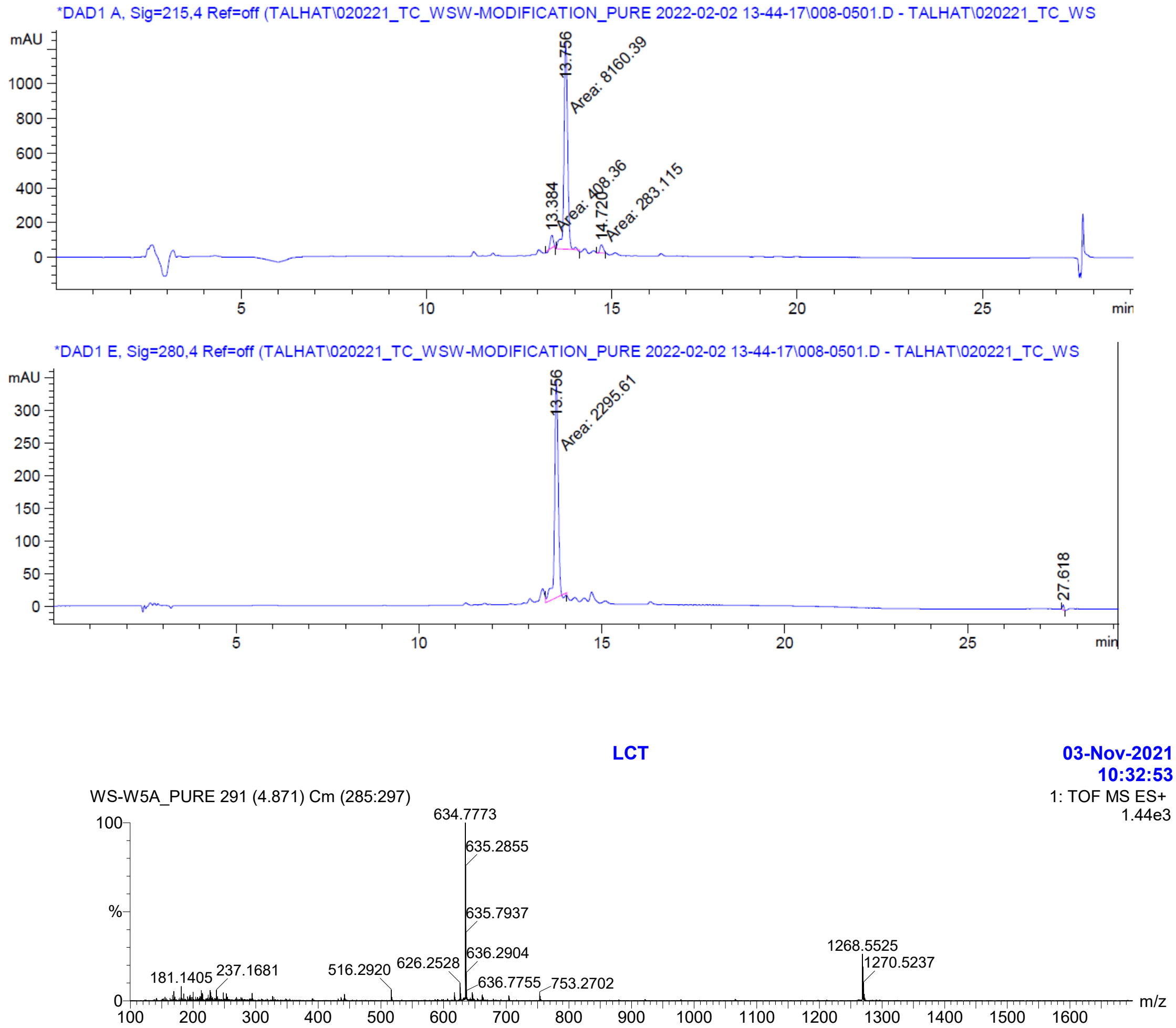

Figure S12. Peptide **12** (FITC-Ahx- SYPG(Cha)SW) purification data. (A) Peptide purity 92%. *Top*, HPLC spectra at 215 nm retention time 13.756 minutes. *Bottom*, HPLC spectra at 280 nm retention time 13.756 minutes. (B) LC-MS data. Extracted mass spectra showed expected mass-to-charge ratios: m/z 1268 $[M+H]^+$ and 634 $[M+2H]^+$.

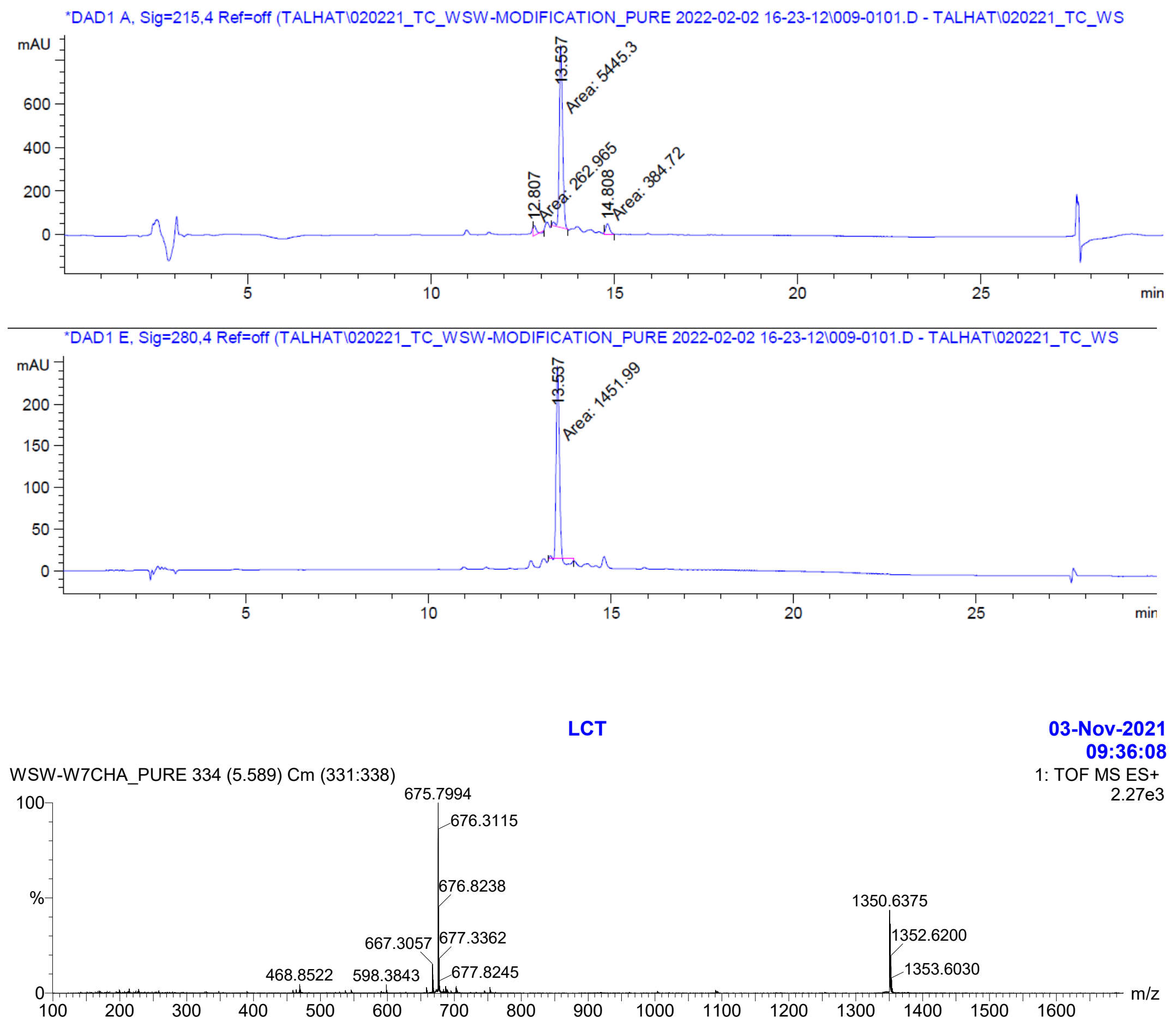

Figure S13. Peptide **13** (FITC-Ahx- SYPGWS(Cha)) purification data. (A) Peptide purity 90%. *Top*, HPLC spectra at 215 nm retention time 13.756 minutes. *Bottom*, HPLC spectra at 280 nm retention time 13.756 minutes. (B) LC-MS data. Extracted mass spectra showed expected mass-to-charge ratios: m/z 1350 $[M+H]^+$ and 675 $[M+2H]^+$.

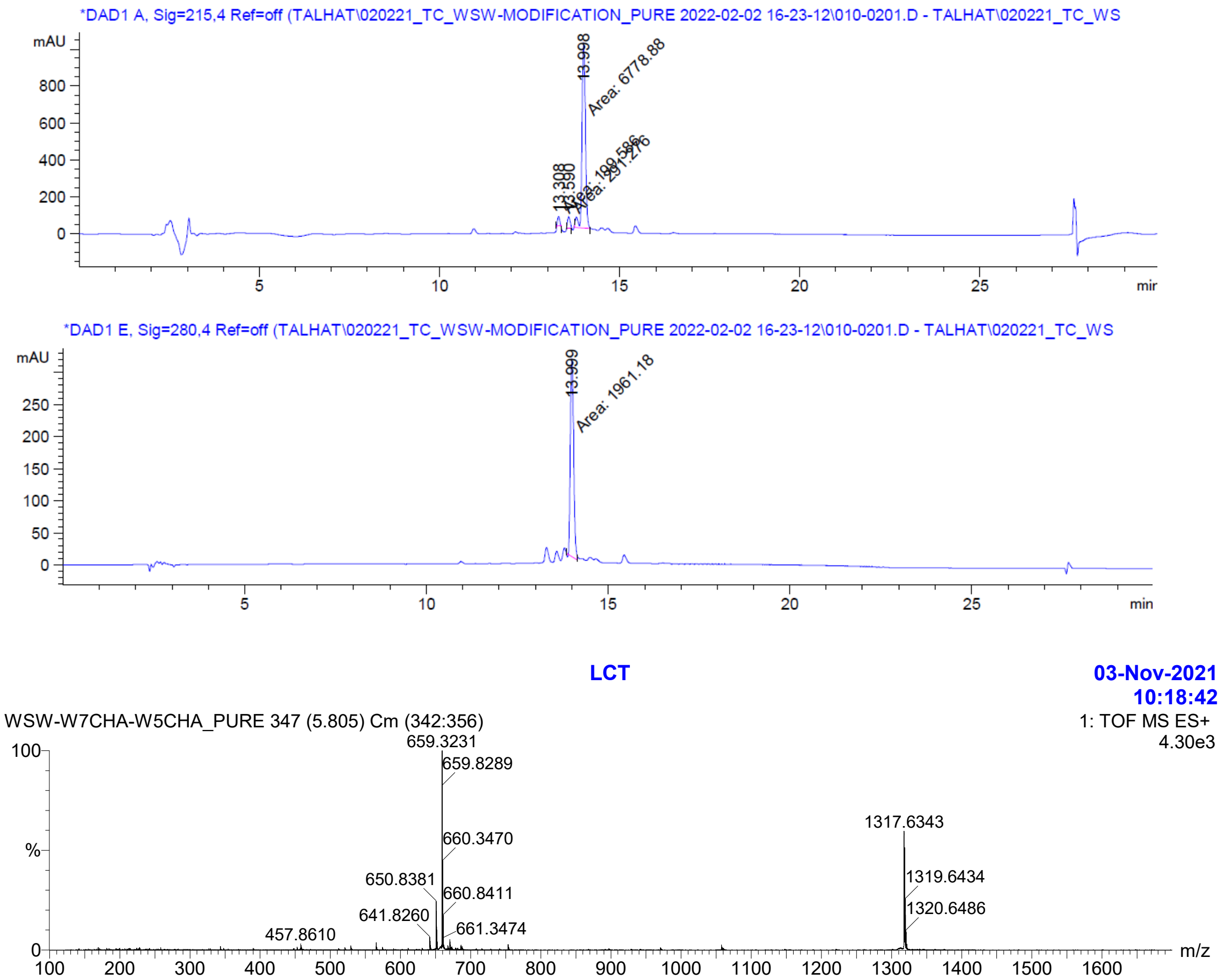

Figure S14. Peptide **14** (FITC-Ahx-SYPG(Cha)S(Cha)) purification data. (A) Peptide purity 93%. *Top,* HPLC spectra at 215 nm retention time 13.998 minutes. *Bottom*, HPLC spectra at 280 nm retention time 13.998 minutes. (B) LC-MS data. Extracted mass spectra showed expected mass-to-charge ratios: m/z 1317 [M+H]+ and 659 [M+2H]+.

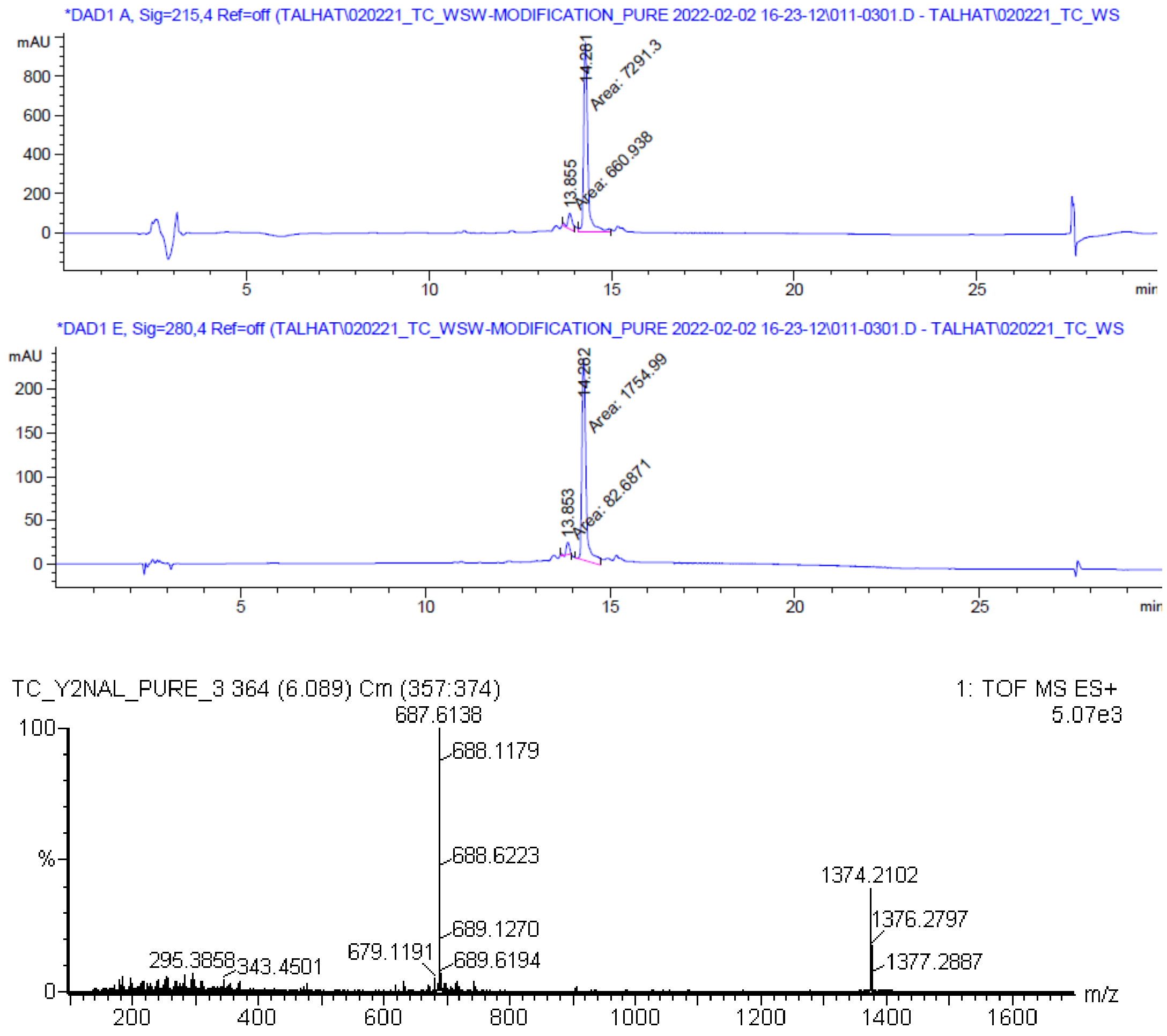

Figure S15. Peptide **15** (FITC-Ahx- S(Nal)PGWSW) purification data. (A) Peptide purity 92%. *Top*, HPLC spectra at 215 nm retention time 14.282 minutes. *Bottom,* HPLC spectra at 280 nm retention time 14.282 minutes. (B) LC-MS data. Extracted mass spectra showed expected mass-to-charge ratios: m/z 1374 $[M+H]^+$ and 687 $[M+2H]^+$.

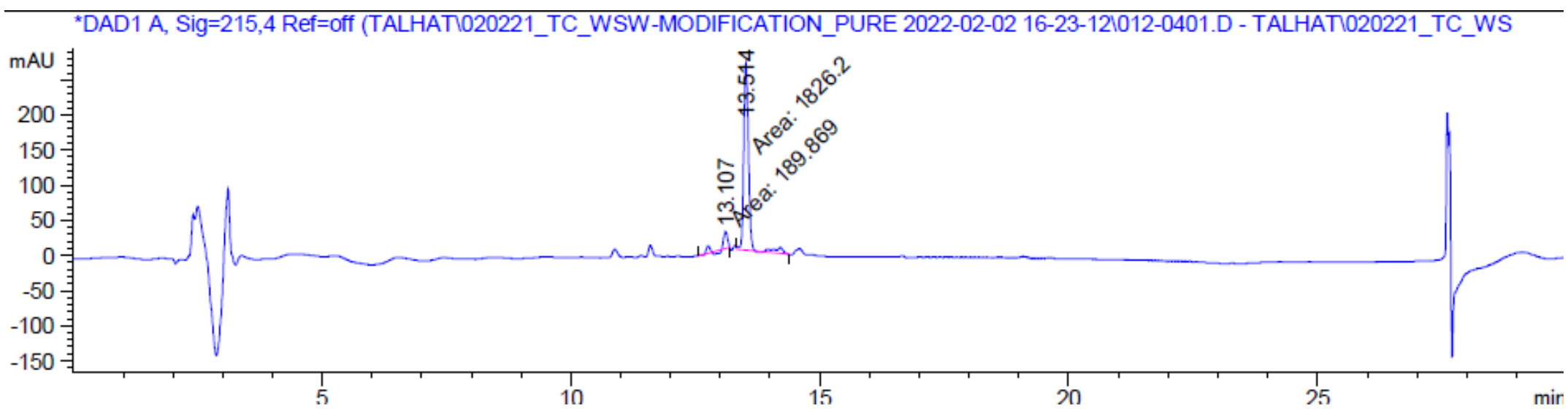

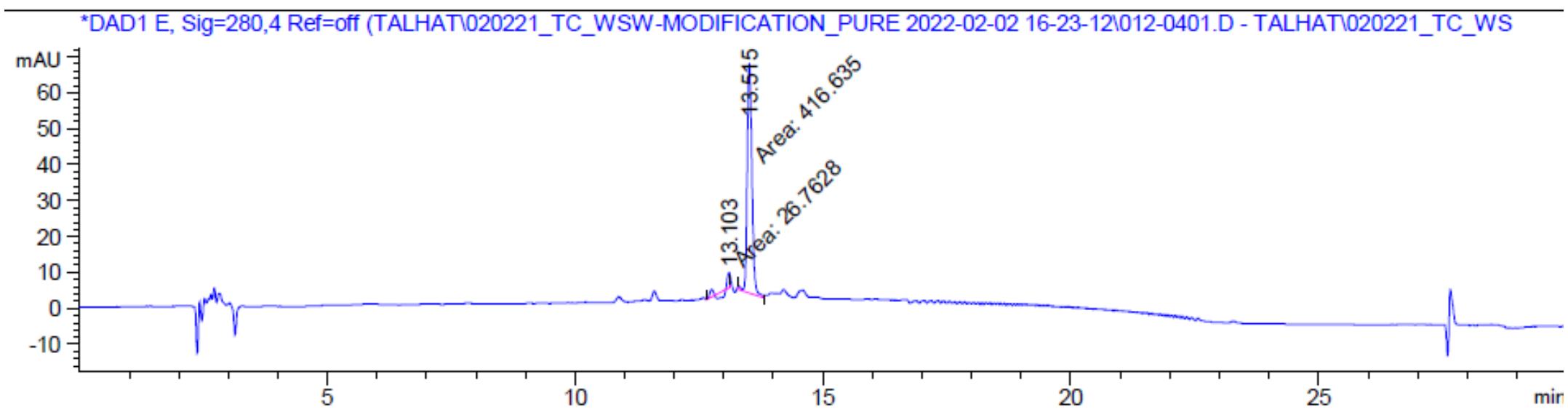

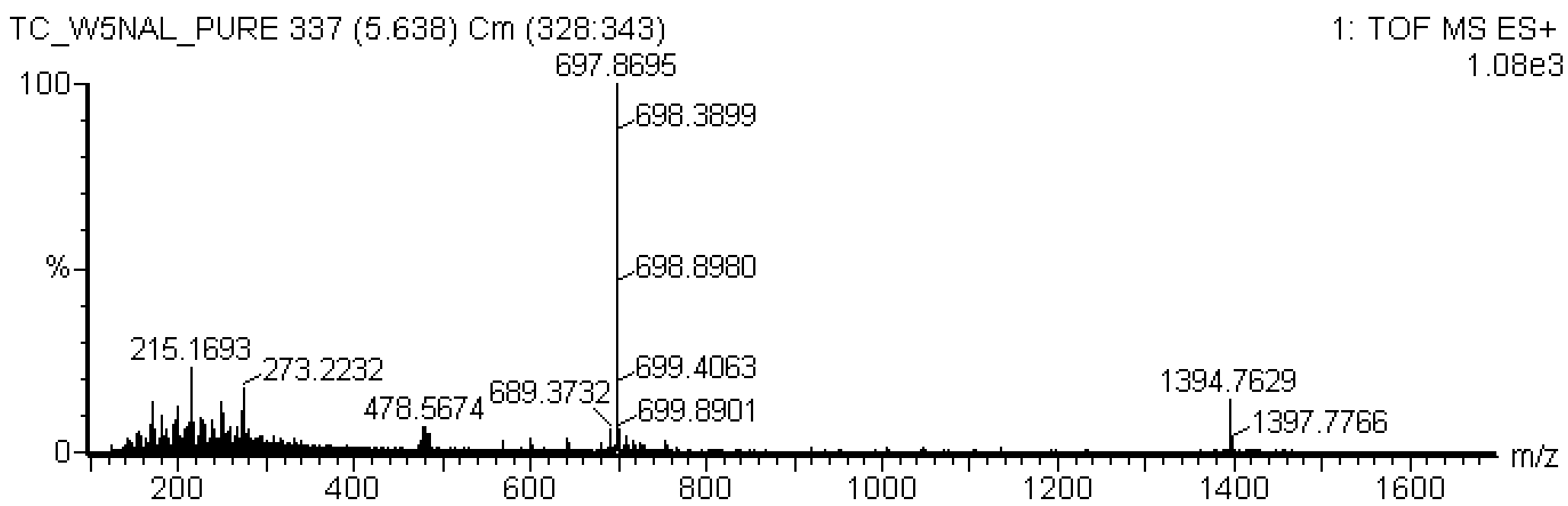

Figure S16. Peptide **16** (FITC-Ahx- SYPG(Nal)SW) purification data. (A) Peptide purity 91%. *Top*, HPLC spectra at 215 nm retention time 13.314 minutes. *Bottom*, HPLC spectra at 280 nm retention time 13.315 minutes. Figure S **Error! No text of specified style in document.**.1: Peptide 34. LC-MS data for W5Cha (FITC-Ahx-SYPG(Nal)SW). Extracted mass spectra showed expected mass-to-charge ratios: m/z 1394 $[M+H]^+$ and 697 $[M+2H]^+$.

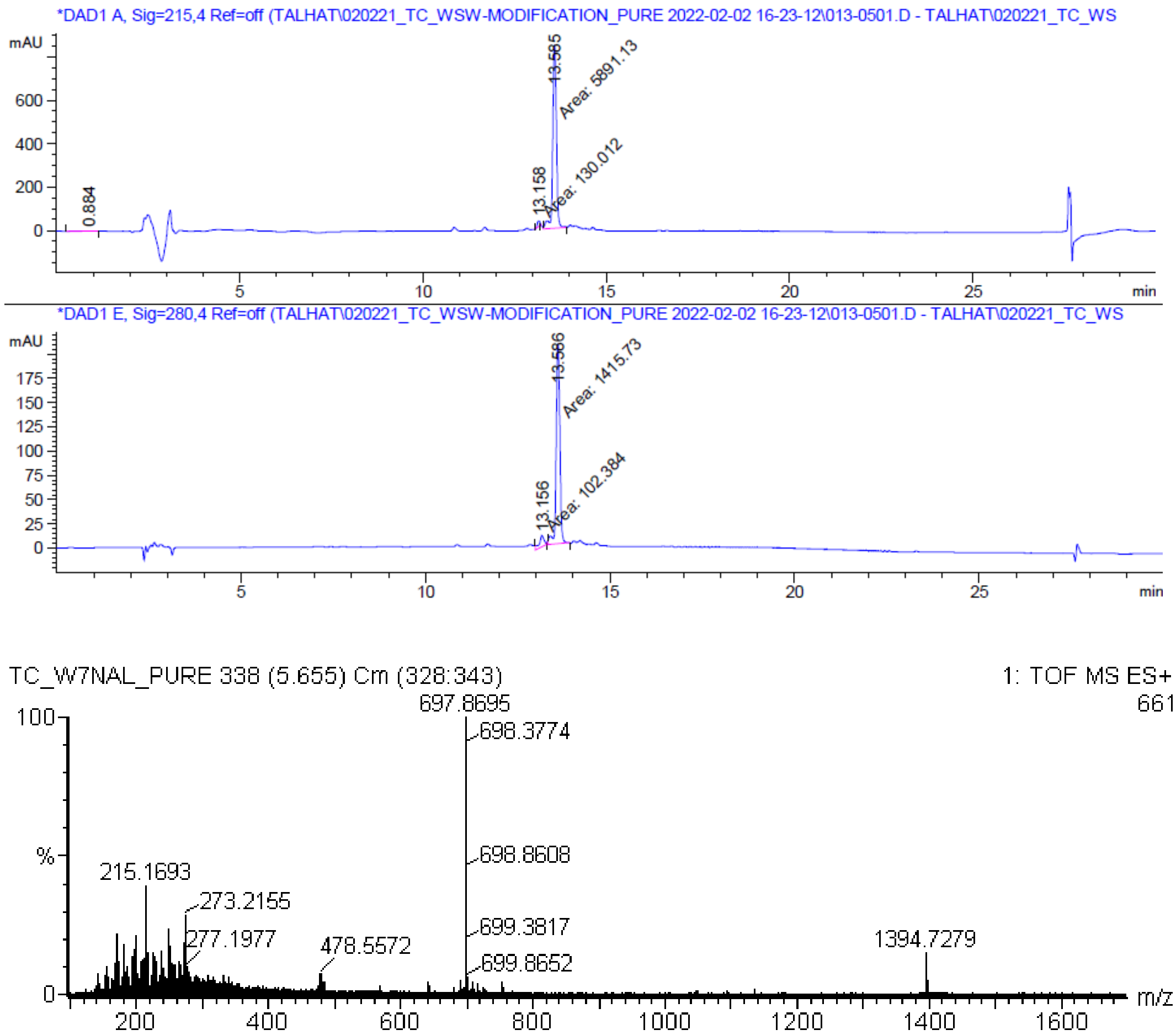

Figure S17. Peptide **17** (FITC-Ahx- SYPGWS(Nal)) purification data. (A) Peptide purity > 95%. *Top*, HPLC spectra at 215 nm retention time 13.585 minutes. *Bottom*, HPLC spectra at 280 nm retention time 13.586 minutes. (B) LC-MS data. Extracted mass spectra showed expected mass-to-charge ratios: m/z 1394 $[M+H]^+$ and 697 $[M+2H]^+$.

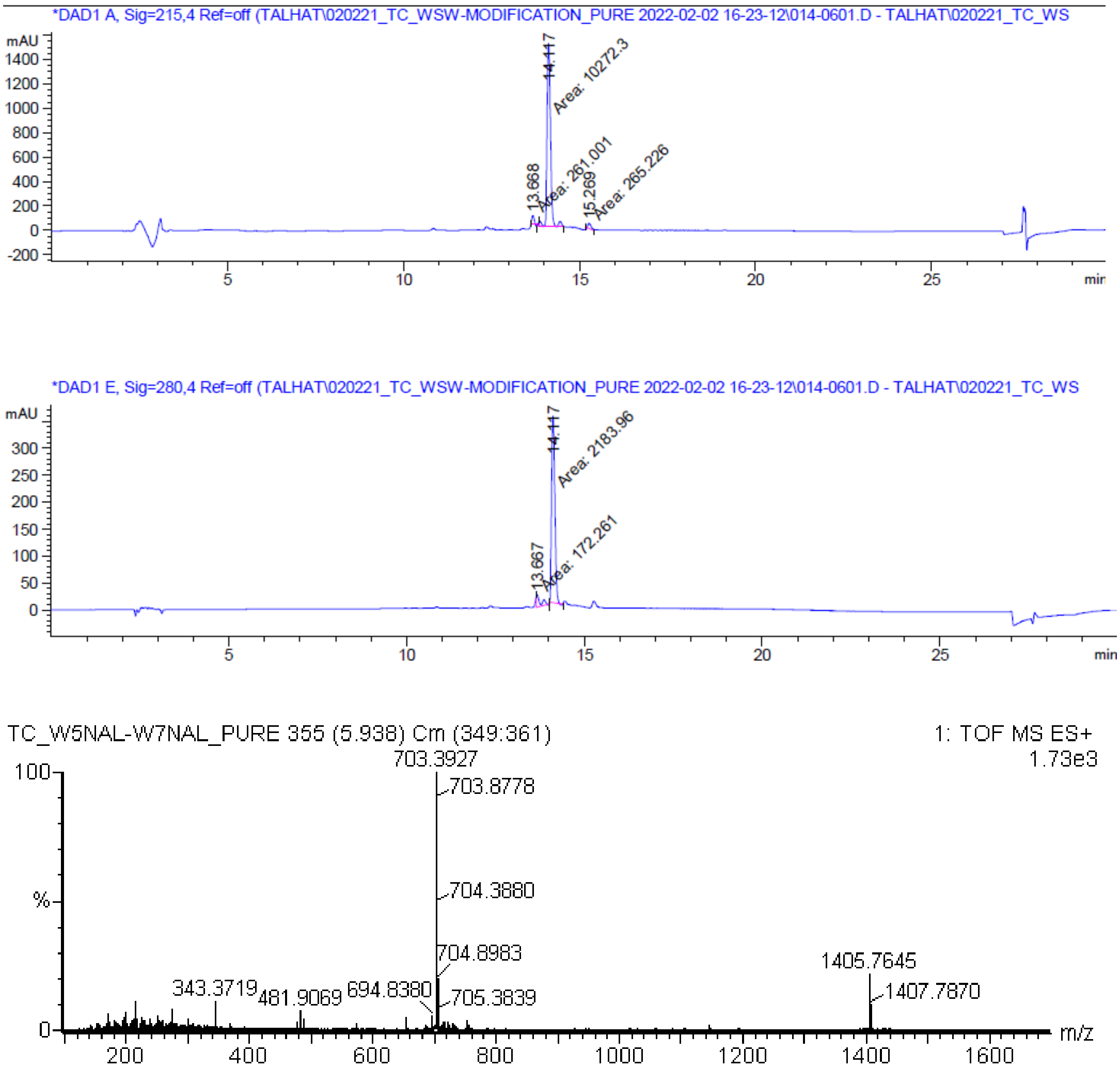

Figure S18. Peptide **18 (**FITC-Ahx-SYPG(Nal)S(Nal)) purification data. (A) peptide purity > 95%. Top HPLC spectra at 215 nm retention time 14.117 minutes. Bottom HPLC spectra at 280 nm retention time 14.117 minutes. (B) LC-MS data. Extracted mass spectra showed expected mass-to-charge ratios: m/z 1405 $[M+H]^+$ and 703 $[M+2H]^+$.

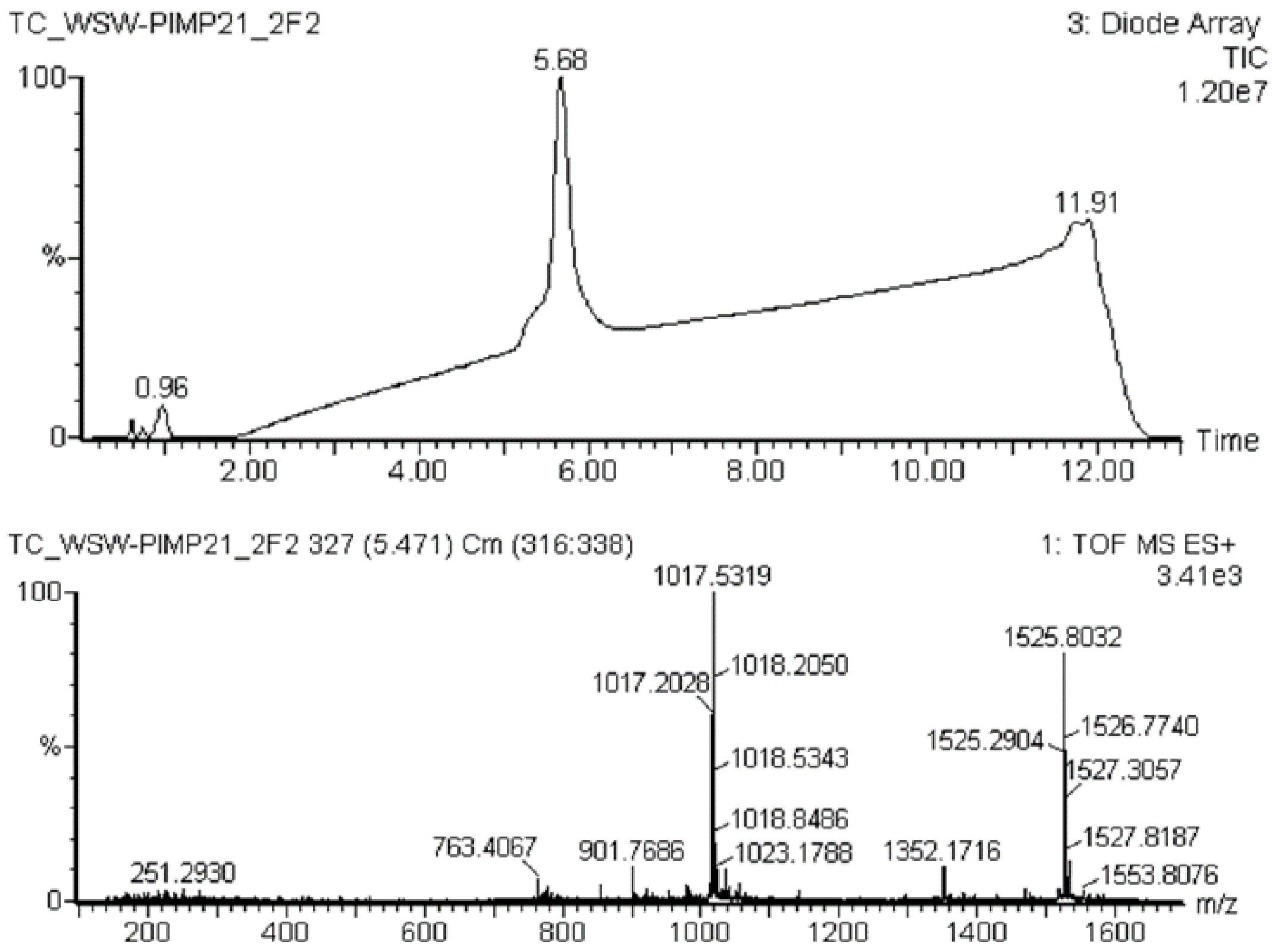

Figure S19. Peptide **20** (SYPGWSW-Ahx-K(FITC)-Ahx-ALWPPNLHAWVP) purification data. (A) HPLC spectra. (B) LC-MS data. (B) Analysis of peaks (α,β) showed expected mass-to-charge ratios: m/z 1525.8032 $[M+2H]^+$ and 1017.5319 $[M+3H]^+$.

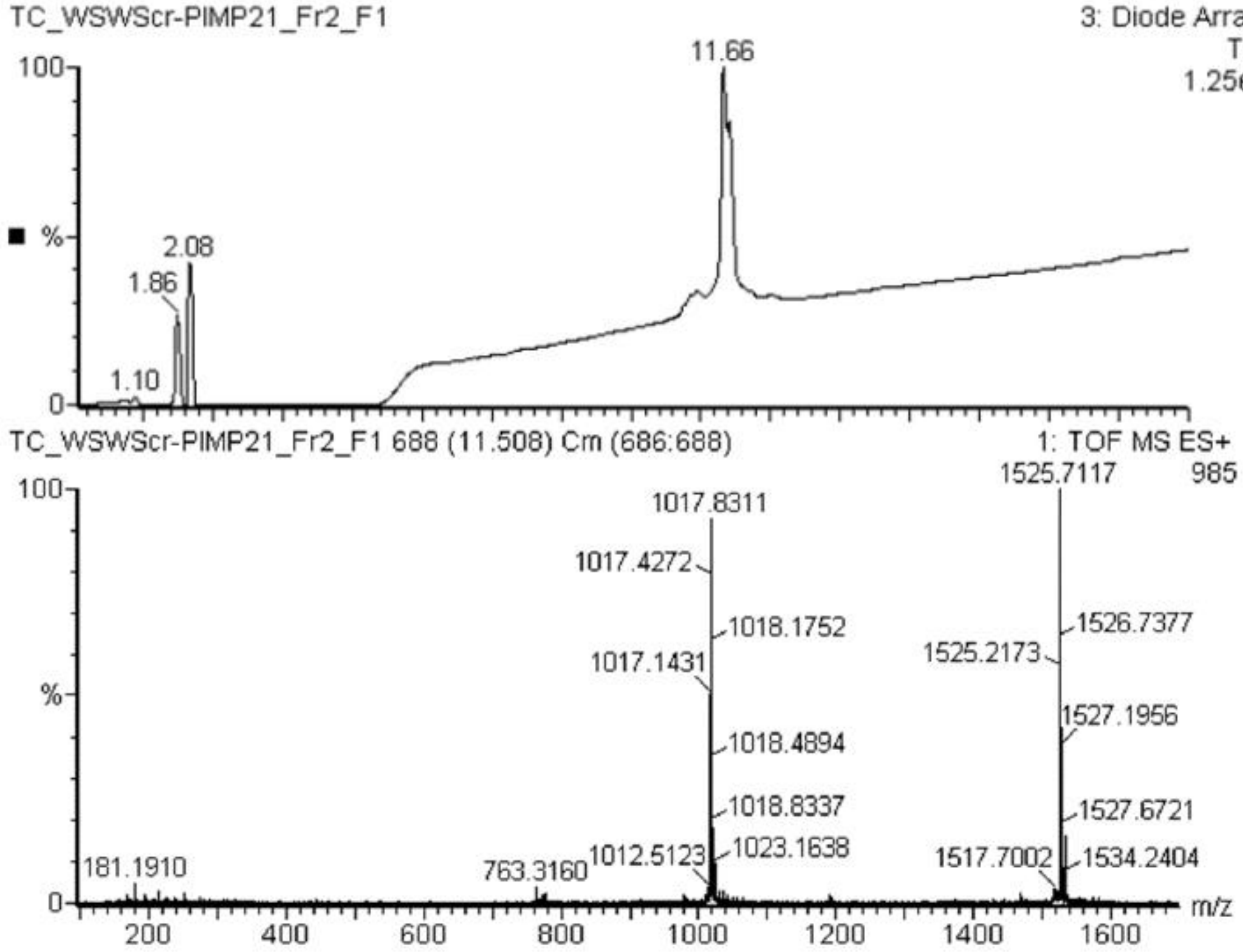

Figure S20. Peptide **21** (SYPGWSW-Ahx-K(FITC)-Ahx-LPPAPWNWAHVL) purification data. (A) HPLC spectra. (B) LC-MS data. (B) Analysis of peaks (α,β) showed expected mass-to-charge ratios: m/z m/z 1525.7117 $[M+2H]^+$ and 1017.8311 $[M+3H]^+$.

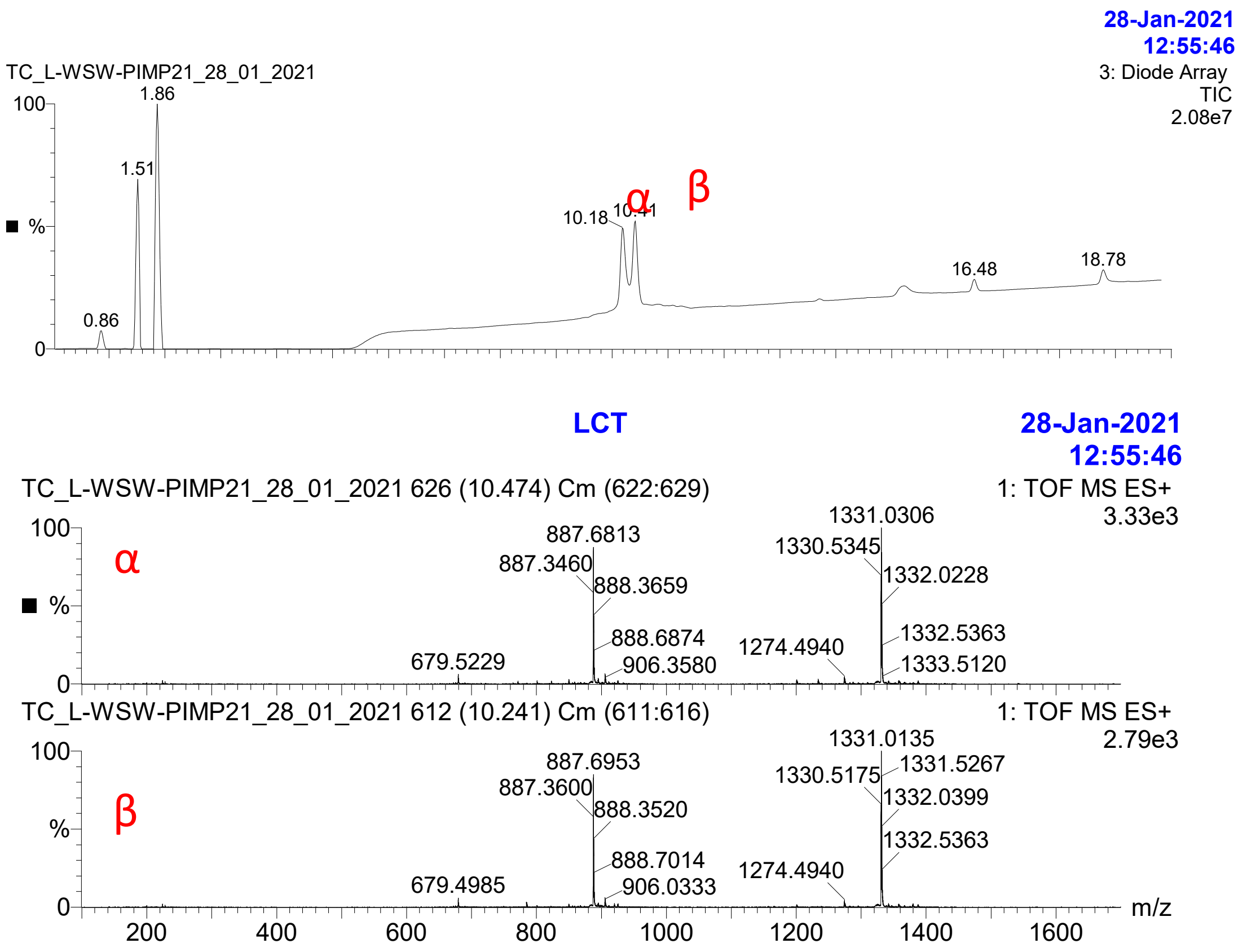

Figure S21, Peptide **22** (SYPGWSW-Ahx-K-Ahx-ALWPPNLHAWVP) purification data. (A) HPLC spectra. (B) Analysis of peaks (α,β) showed expected mass-to-charge ratios: m/z 1331 $[M+2H]^+$ and 887 $[M+3H]^+$ for both peaks, indicating the potential presence of conformers.

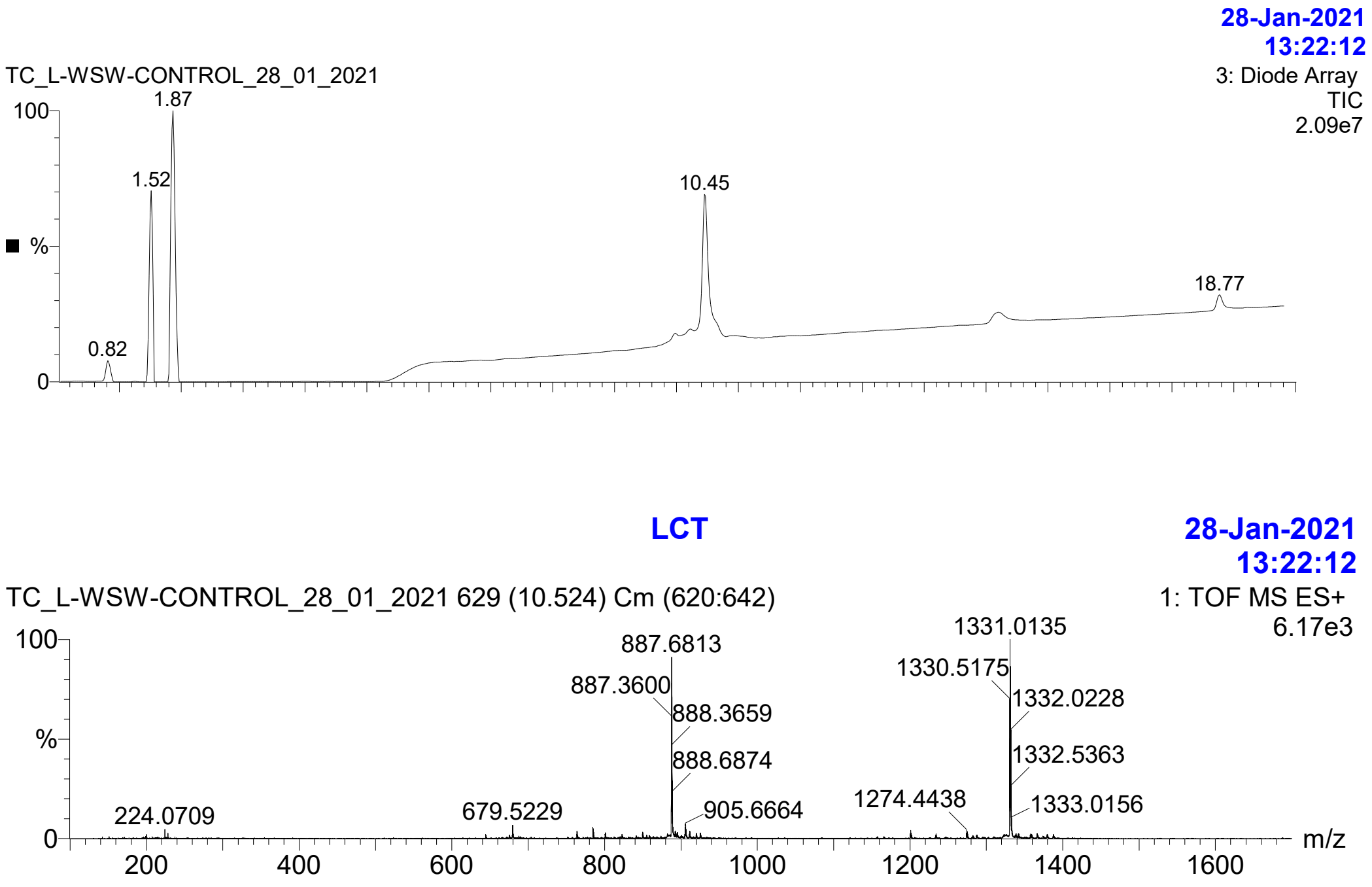

Figure S22. Peptide **23** (SYPGWSW-Ahx-K-Ahx-LPPAPWNWAHVL) purification data. (A) HPLC spectra. (B) Analysis of peaks (α,β) showed expected mass-to-charge ratios: m/z 1331 $[M+2H]^+$ and 887 $[M+3H]^+$.
